# The SWI/SNF-Related, Matrix Associated, Actin-Dependent Regulator of Chromatin A4 Core Complex Represses Respiratory Syncytial Virus-Induced Syncytia Formation and Subepithelial Myofibroblast Transition

**DOI:** 10.3389/fimmu.2021.633654

**Published:** 2021-03-01

**Authors:** Xiaofang Xu, Dianhua Qiao, Chenyang Dong, Morgan Mann, Roberto P. Garofalo, Sunduz Keles, Allan R. Brasier

**Affiliations:** ^1^Department of Internal Medicine, University of Wisconsin-Madison School of Medicine and Public Health (SMPH), Madison, WI, United States; ^2^Department of Statistics, University of Wisconsin-Madison, Madison, WI, United States; ^3^Department of Pediatrics, University of Texas Medical Branch, Galveston, TX, United States; ^4^Department of Biostatistics & Medical Informatics, University of Wisconsin-Madison, Madison, WI, United States; ^5^Institute for Clinical and Translational Research (ICTR), University of Wisconsin-Madison, Madison, WI, United States

**Keywords:** epithelial mesenchymal transition, matrix metalloproteinase (MMP), myofibroblast transition, airway remodeling, extracellular matrix

## Abstract

Epigenetics plays an important role in the priming the dynamic response of airway epithelial cells to infectious and environmental stressors. Here, we examine the epigenetic role of the SWI/SNF Related, Matrix Associated, Actin Dependent Regulator of Chromatin A4 (SMARCA4) in the epithelial response to RSV infection. Depletion of SMARCA4 destabilized the abundance of the SMARCE1/ARID1A SWI/SNF subunits, disrupting the innate response and triggering a hybrid epithelial/mesenchymal (E/M) state. Assaying SMARCA4 complex-regulated open chromatin domains by transposase cleavage -next generation sequencing (ATAC-Seq), we observed that the majority of cleavage sites in uninfected cells have reduced chromatin accessibility. Paradoxically, SMARCA4 complex-depleted cells showed enhanced RSV-inducible chromatin opening and gene expression in the EMT pathway genes, *MMP9, SNAI1/2, VIM*, and *CDH2*. Focusing on the key MMP9, we observed that SMARCA4 complex depletion reduced basal BRD4 and RNA Polymerase II binding, but enhanced BRD4/Pol II binding in response to RSV infection. In addition, we observed that MMP9 secretion in SMARCA4 complex deficient cells contributes to mesenchymal transition, cellular fusion (syncytia) and subepithelial myofibroblast transition. We conclude the SMARCA4 complex is a transcriptional repressor of epithelial plasticity, whose depletion triggers a hybrid E/M state that affects the dynamic response of the small airway epithelial cell in mucosal remodeling *via* paracrine MMP9 activity.

## Introduction

The human airway is lined with a contiguous mucosal surface composed of highly differentiated airway epithelial cells that play important role in gas exchange, maintenance of fluid balance, control of vascular reactivity and initiation of the innate response (1). Ultrastructural, biochemical, and cell-fate mapping studies have identified at least 8 common epithelial phenotypes that differ in secretory, self-renewal, mucociliary clearance properties (2), populating distinct zones of the airway, and playing unique roles in pulmonary disease (3). In particular, environmental oxidant exposure or viral infection of the lower airway cuboidal epithelial cell population triggers an injury-repair process that participates in chronic obstructive pulmonary disease (4, 5) and viral bronchiolitis (6). Understanding the mechanisms controlling this epithelial injury-repair process will be critical to developing new treatments for these important diseases.

Although the resting airway epithelium normally slowly repopulates (once every 30–50 d), mucosal damage triggers a rapid injury response. Within minutes of mucosal denudation, nearby epithelial cells de-differentiate, enabling them to migrate to repopulate the injured area (7). This epithelial injury-repair process is a sterotypic genomic response that results in loss of epithelial differentiation features [cadherin (CDH1)] and acquisition of mesenchymal properties [vimentin (VIM) and extracellular matrix (ECM)-remodeling matrix metalloproteinases (MMP)]. This genomic reprogramming is known as “type II” (non-oncogenic) epithelial-to-mesenchymal transition (EMT) (8, 9). In regions of the airway undergoing EMT, the mucosal barrier is disrupted and lamina reticularis is restructured. Systems-levels studies have shown that EMT involves a series of reversible cell-state transitions [Reviewed in (10)]. The first step in EMT is loss of cell surface CDH1, mediated by post-transcriptional regulation (11), producing a partial EMT (pEMT) state associated with expression of epithelial (E) and mesenchymal (M) genes. With continued stress, growth factor stimulation, and changes in the ECM tensile strength, cells in this metastable E/M state express mesenchymal intermediate filaments and core transcription factors (ZEB, SNAI1), resulting in a largely irreversible, fully committed mesenchymal phenotype (12). Cellular reprogramming produced by EMT disrupts mucosal innate immunity (13, 14) and promotes ECM deposition, characteristic of obstructive lung diseases.

Respiratory syncytia virus (RSV) is one of the most common causes of lower respiratory tract infection (LRTI) in infants and young children, responsible for the majority of pediatric hospitalizations (15). RSV replicates primarily in the airway epithelial cell (16), a process that disrupts cellular morphology and causes formation of syncytia that protrude and slough into the infected airway, contributing to luminal obstruction, ventilation-perfusion mismatching and hypoxia (16–18). Severe LRTIs are important because these may be linked to the development of atopy, asthma and chronic obstructive lung disease (19).

Viral infections in early life are associated with phenotypic changes of the structural cells of the airway (20), including expansion of the basement membrane [aka, “remodeling” (21)]. Recent work has implicated a central role of the innate immune response effector, NFκB, in coupling inflammation and cellular reprogramming (13, 22, 23). RNA sequencing studies have shown that NFκB is a master transcription factor of EMT (23, 24) and that NFκB activation binds to (25, 26)- and positions the atypical histone acetyltransferase, bromodomain containing protein 4 (BRD4) to form “super enhancers” controlling cell-identity genes (27) and to trigger expression of remodeling factors *via* transcriptional elongation (28). Inhibition of NFκB or BRD4 blocks innate response and growth factor-induced EMT (23, 29) and remodeling (30). These studies indicate that BRD4 and other chromatin regulators play important roles in the innate response and cellular reprogramming. The identity and role of these remodeling factors are not fully understood.

Previous unbiased protein-protein interaction studies showed that BRD4 interacts with SMARCA4/Brg1, a central catalytic core of the ATP-dependent SWItch/sucrose-nonfermentable (SWI/SNF) chromatin remodeling complex (31). SMARCA4 complexes control gene networks important in cell morphological change, cell identity determination, cellular adhesion and metastasis. In this study, we observed that RSV also induces SMARCA4 expression and explored its role in the cellular response to infection. We found that SMARCA4 knockdown (KD) leads to unscheduled expression of a hybrid E/M program, characterized by a dramatic loss of cell-surface CDH1, enhanced expression of a core of EMT regulators, *MMP9, VIM*, and *SNAI*, and expression of MET counter-regulators, *CDH3* and *ESRP1*. Reasoning that SMARCA4 mediated chromatin accessibility of EMT regulators, we conducted ATAC-Seq experiments, which revealed a global loss of chromatin accessibility in uninfected SMARCA4-KD cells, yet chromatin accessibility was upregulated over that of WT in response to RSV infection. Focusing on the mechanisms controlling MMP9, we observe that the core transcriptional elongation complex, BRD4/Pol II show enhanced loading and inducibility in response to RSV infection in the SMARCA4-KD cells. MMP9 mediates the initial phase of EMT transition by destabilizing the cell surface CDH1 expression and promoting cellular fusion in response to RSV. Finally, in an epithelial-lung fibroblast coculture model, we observed enhanced myofibroblast transdifferentiation that was potentiated by RSV infection and MMP-9 dependent. These data indicate SMARCA4 is a transcriptional repressor of a “default” pEMT program and affects the dynamic response of the small airway epithelial cell *via* paracrine MMP9 activity mediating virus-inducible remodeling.

## Materials and Methods

### Cell Culture and Knockout Cell Lines Generation

Human small airway epithelial cells (hSAEC) were grown either on plastic culture ware or on transwell polyester membrane (Corning, 3460, co-culture experiment) in SAGM small airway epithelial cell growth medium (Lonza, cc-3118). Human primary lung fibroblast was from ATCC (PCS-201-013) and grown in low-serum (ATCC: PCS-201-041) and serum-free fibroblast growth medium (ATCC: PCS-201-040).

SMARCA4 KD cells with short hairpin RNA (shRNA) were generated using lentiviral particles (Sigma, TRCN0000380723 for SMARCA4 KD and SHC001V for control). Cells were selected and maintained in puromycin (10 µg/ml).

### Preparation of Sucrose Cushion-Purified RSV (pRSV)

The human RSV Long strain was grown in Hep-2 cells and prepared by sucrose cushion centrifugation as described (32). The viral titer was determined by a methylcellulose plaque assay. Sucrose cushion purified RSV (pRSV) aliquots were quick-frozen in dry ice-ethanol, and stored at -70°C until use.

### RNA Isolation and qRT-PCR

Cells were harvested for RNA isolation using RNeasy kit with on-column DNase digestion (Qiagen). Synthesis of complementary DNAs (cDNAs) was done with First Strand cDNA Synthesis Kit (Thermo Scientific). There were two real-time quantitative reverse transcriptase PCR (qRT-PCR) assays used: one was using SYBR Green Master mix (Bio-Rad); the other was with TaqMan primers and TaqMan Fast Advanced Master Mix (Thermo Scientific). The TaqMan assays are shown in **Table 1**; sequences of the SYBR Green PCR primers are given in **Table 2**.

**Table 1:** Primers used for Taqman RT PCR assays.

	Taqman primers
SMARCA4	Hs00231324_m1
MMP9	Hs00957562_m1
SNAI1	Hs00195591_m1
CDH2	Hs00983056_m1
CDH1	Hs01023895_m1
VIM	Hs00958111_m1
PPIA	Hs04194521_s1

**Table 2:** Primers used for Sybr-Green Q-RT-PCR and Q-gPCR assays.

Q-RT-PCR		
Gene	Forward (5’-3’)	Reverse (5’-3’)
*PPI1A*	TTCATCTGCACTGCCAAGAC	TCGAGTTGTCCACAGTCAGC
*RSV-N*	AAGGGATTTTTGCAGGATTGTTT	TCCCCACCGTAACATCACTTG
**Q-gPCR**		
MMP9-promoter	GTGTTGCAAAAGGCCAAGGA	TGGTCTGAAAGCCTCCAGTG
MMP9-enhancer2	TGGGAGGTGTGACTTGGTGT	CCCTAGCTTCAGGGGAATGG
MMP9-enhancer1	AAGGGAGGTGTGGTGGTTCT	AGGTTACACACACCCTGCAA

### Protein Lysate Preparation and Western Blot

Cells were trypsinized, pelleted and washed twice with cold phosphate-buffered saline (PBS). Cell pellets were then lysed in cold low ionic strength buffer (50 mM NaCl, 1% IGEPAL, 10% Glycerol, 10 mM HEPES, pH7.4) with fresh added proteinase inhibitor cocktail (Sigma), 1 mM DTT and 1 mM PMSF (Sigma). 1 µl of cell lysate was taken out for protein measurement (DC protein assay kit, Bio-Rad). Seventy-five micrograms were mixed with 2xSDS sample buffer, sonicated three times for 15 s each time (Branson150), centrifuged at 10,000 rpm for 20 min and supernatants were denatured at 95°C for 5 minutes. Proteins were resolved using Criterion TGX 4%–15% precast gel and transferred to nitrocellulose membrane with Bio-Rad Trans-Blot Turbo transfer system. Primary antibodies used were anti-SMARCA4 (Cell Signaling, 52251; LSBio, LS-C413011); anti-SMARCA2(Cell Signaling, 11966); anti-ARID1A (Cell Signaling 12354); SMARCE1 (Cell Signaling 33360); anti-TBP(Abcam, ab818); and anti-beta Tubulin (Abcam, ab6046).

### Immunofluorescence Microscopy

hSAECs and SMARCA4 KD cells were plated on coverslips or transwell membrane. Human primary fibroblasts were plated on coverslips in the lower compartment of transwell chamber. After virus infection, with or without 5 µM MMP-9 inhibitor I (Santa Cruz, CAS 1177749-58-4), cells were fixed with 4% paraformaldehyde, permeablized with 0.1% Triton X-100, blocked with 5% goat serum and incubated with primary antibody overnight. Primary antibodies used were anti-Vimentin (Sigma, V6630); anti-E-Cadherin (cell Signaling, 3195); anti-Ki67 (Thermo, MA5-14520). On second day, Alexa-fluor goat secondary antibody and/or phalloidin (Thermo, A12380) were added. After 1 h, cells were washed and mounted using Prolong Diamond Antifade Mountant with 4’,6-diamidino-2-phenylindole (DAPI, Molecular Probes). To assay syncytia formation, fixed cells were stained with CellBrite (Biotium, 30090, 5 ul/1ml PBS), and mounted with Antifade Mountant containing DAPI. The cells were visualized with ECHO Revolve fluorescent microscope.

### RNA-Next Generation Sequencing

Total RNA was isolated using RNeasy kit with on-column DNase I digestion (Qiagen). RNA was quantified (Nanodrop) and integrity verified (Agilent) prior to sequencing. Libraries were produced by the TruSeq Stranded mRNA. The 24 separate RNA samples (N=4 each for hSAEC and SMARCA4 KD cells at time 0, 16 h, and 24 h of RSV treatment) were bar-coded and subjected to Illumina HiSeq 2000 paired-end sequencing. The trimming software skewer was used to process raw fastq files and QC statistics. The trimmed paired-end reads were aligned against human genome hg38 using Salmon. Mapped paired-end reads for both genes and transcripts (isoforms) were counted in each sample using RSEM. Contrasts were compared for genotypes and treatment conditions using DESeq2 (33). Hierarchial Clustering (HC) was performed to identify patterns in gene expression in the complexHeatMap program in R (version 1.0.12). Gene Set Enrichment Analysis (GSEA) and Hierarchial Clustering (HC) from DEG were performed to detect enrichment of biological pathways and gene regulatory networks.

### Assay for Transposase-Accessible Chromatin Sequencing

ATAC-seq experiment in hSAEC cells were performed in two biological replicates per treatment (34). Briefly, 50,000 cells (>95% viability) were trypsinized and washed. Cell pellets were re-suspended in 300ul of cold lysis buffer (10 mM Tris-HCl, pH7.4, 10 mM NaCl, 3 mM MgCl2, 0.1% IGEPAL CA-630) and incubate on ice for 10 min. Nuclei were pelleted, re-suspended in 50 µl of transposition mixture (25 µl 2x TD buffer (Illumina, 15027866), 2.5 µl TDE1 (Illumina Nextera Tn5 transposase, 15027865), 22.5 µl nuclease-free water) and incubated for 30 min at 37°C water bath with gentle mixing. After cleaning with Qiagen MinElute PCR purification kit (Qiagen, 28204), eluted DNA was pre-amplified for five cycles in PCR mix (30 µl NEBNext High-Fidelity 2x master mix (NEB, M054 1S), 10 µl primer mix (Illumina, NEXtera DNA UD indexes20026121), 20 µl purified DNA) for five cycles. Additional three amplification cycles were added based on QPCR amplification. PCR reactions were then purified with Qiagen PCR purification kit and sent to University of Wisconsin Biotechnology Center. Libraries were further purified, quantified and analyzed on a bioanalyzer and sequenced on a NovaSeq 6000 with 50 million reads per sample.

### ATAC-Seq Data Analysis

Alignment of ATAC-seq reads. Illumina Nextera adapters were trimmed with cutadapt (version 2.0) (35) with option “-q 30 –minimum-length 36”. Paired-end ATAC-seq reads were aligned to the human genome assembly (hg19) with bowtie2 (version 2.3.4.1) (36) with option “-X 800 –no-mixed –no-discordant”. For each sample, unmapped reads were filtered out by using SAMtools view (version 1.8) (37) with option “-F 4” and mitochondrial reads were removed. Duplicated reads were removed with Picard tools (version 2.9.2) (38). We had on average 51.4 ± 8.2 million reads per sample.

Identification of reproducible ATAC-seq peaks and differential accessibility analysis. Peaks were called for pooled and individual samples by MACS2 (39). The final 27,779 master peaks (mean width 988.8 ± 420.5 bp) were generated from the corresponding pooled sets with irreproducible discovery rate (IDR) analysis (40) at IDR of 0.05. We utilized “getCounts()” function in chromVAR (41) to obtain the count matrix of Tn5 cuts. We then performed differential accessibility analysis with DESeq2 (33) with sequencing depth/1e8 as the size factor. We first determined 20,724 up-regulating and 42 down-regulating peaks under RSV stimulation (FDR of 0.05, fold change of accessibility > 2). We then identified 9,374 wild type specific peaks and four for the knockdown type (FDR of 0.05, fold change of accessibility > 2). The genomic compartment annotations were obtained using ChIPseeker (42).

Motif enrichment analysis. For 7,326 RSV up-regulating and 3,763 wild type specific promoter peaks, we performed motif enrichment analysis on these peaks with MEME-CHIP (43) with respect to JASPAR motif library (44).

### Two-Step Chromatin IP (XChIP)

Triplicate wells of hSAECs in 60 mm dishes were washed twice with phosphate-buffered saline. Protein–protein cross-linking was first performed with DSG (2 mM, 45 min at 22°C) followed by protein–DNA cross-linking with formaldehyde as previously described (45). Equal amounts of sheared chromatin were immunoprecipitated (IPed) overnight at 4°C with 4 µg indicated Ab in ChIP dilution buffer. IPs were collected with 40 μl protein-A magnetic beads (Dynal Inc), washed and eluted in 250 µl elution buffer for 15 min at room temperature.

### Quantitative Genomic PCR (Q-gPCR)

Gene enrichment in XChIP was determined by Q-gPCR as previously described using region-specific PCR primers (**Table 2**). The fold change of DNA in each IP was determined by normalizing the absolute amount to input DNA reference and calculating the fold change relative to that amount in unstimulated cells.

### Zymography

Cell culture supernatant was collected from scrambled shRNA, SMARCA4 KD, and MMP-9 Inhibitor 1-treated SMARCA4 KD cells in the absence or presence of RSV infection (MOI 1, 24 h). The volume was normalized to cell number and subjected to electrophoresis in a SDS-polyacrylamide gel copolymerized with gelatin. After electrophoresis, SDS was removed from the gel by soaking in 2.5% Triton X-100, 50 mM Tris-HCl (pH 7.5), 5 mM CaCl_2_, and 1 µM ZnCl_2_ for 30 min twice. The gel was incubated a reaction buffer (1% Triton X-100, 50 mM Tris-HCl (pH7.5), 5 mM CaCl_2_ and 1 µM ZnCl_2_), at 37°C for 24 h followed by Coomassie Blue staining. Discolored bands in the gel were visualized to quantify gelatinase activity. The gel image was inverted and quantitated by Image J software.

### Fluorogenic MMP9 Assay

Twenty microliters of fresh medium (as negative control) or conditioned media was combined with 180 µL of MMP reaction buffer (200 mM NaCl, 50 mM Tris-HCl (pH7.6), 5 mM CaCl_2_, 20 µM ZnCl_2_, 0.05% Tween 20) and 10 uM fluorogenic MMP1/MMP9 substrate (Cat. 444221, Millipore, MA) with or without 5 µM MMP9 inhibitor 1(Santa Cruz sc-311437) and incubated at 37°C for 1 h. Fluorescence intensity was measured at a fluorescence plate reader at excitation of 365 nm/emission 450 nm.

### Statistical Analysis

Statistical analysis of the RNA-seq and ATAC-Seq are described earlier in *Materials and Methods* with adjustment for multiple hypothesis testing using False Discovery Rate and Irreproducible Discovery Rates, respectively, as indicated. Nonparametic ATAC seq peak counts were compared using nonparametric Mann-Whitney U test statistic as indicated in the *Results*. Data presented in hierarchical clustering were Z-score normalized across rows and clustered on the basis of Euclidian distance. For Q-RT-PCR and western blots, statistical methods and experimental replicates are given in each *Figure Legend*. Here, two treatment groups were compared using a two-tailed T test. A p value <0.05 was considered significant. For two group comparisons, a two-way ANOVA was performed, followed by Tukey’s *post hoc* test to determine significant differences between groups. Data are plotted as 25-75% interquartile range.

## Results

### Induction of SMARCA4 in Response to RSV

**Figure 1:**
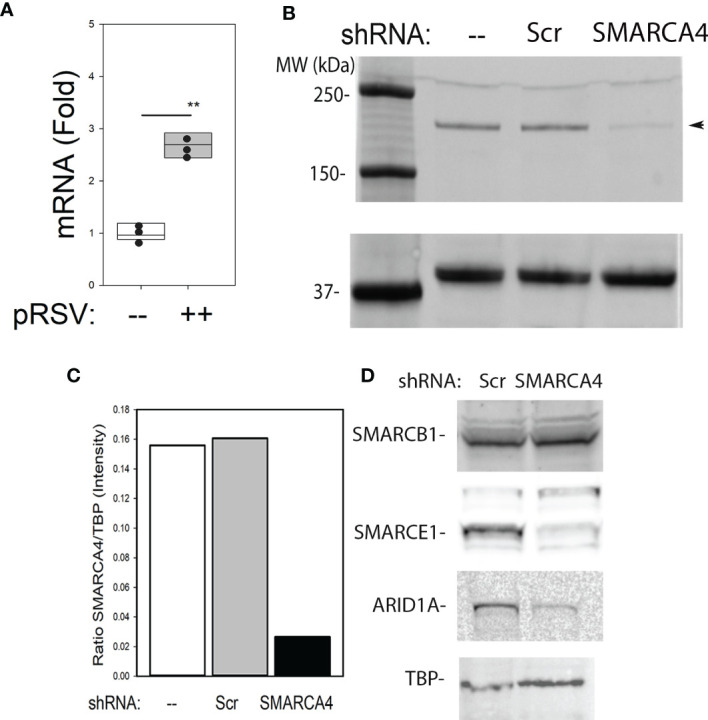
Induction and disruption of the SMARCA4 complex. **(A)** Wild type (WT) human small airway epithelial cells (hSAECs) were mock or pRSV infected (MOI=1). Q-RT-PCR was performed for SMARCA4 and PPIA mRNAs. Shown is fold change of SMARCA4 mRNA normalized to PPIA. **p < 0.01, 2 tailed T test. **(B)** Western immunoblot of SMARC A4 in WT, CON, and SMARCA4-shRNA expressing hSAECs. Left, molecular weight markers (in kDa). TATA binding protein (TBP) is internal control. **(C)** Quantification of individual western blot lanes in **(B)**. Fluorescence intensity was quantified using ImageJ and presented as ratio of SMARCA4/TBP (arbitrary fluorescence units). Reproduced in three biological replicates. **(D)** Effect of SMARCA4 depletion on SWI/SNF core complex. Western blot of SMARCE1, SMARCB1, and ARID1A. TBP is internal loading control. Abundance of SMARCE1 and ARID1A were depleted by 50%, reproduced in n=3 experiments.

We selected highly differentiated *Scgb1a1*-expressing small human airway epithelial cells (hSAECs) for this study because hSAECs are a site of RSV replication in LRTI (46); inhibition of the innate pathway in this cell type *in vivo* blocks RSV-induced airway inflammation and airway obstruction (6); and, *Scgb1a1*^+^ hSAECs produce pathogenic mucin- and T helper 2 lymphocyte-activating cytokines that mediate disease (47). Wild type (WT) hSAECs were infected with sucrose-purified RSV [multiplicity of infection (MOI) =1] for 24 h, and SMARCA4/Brg1 transcripts were quantified by Q-RT-PCR. We observed a 2.8-fold increase (p<0.01, 2-tailed t test, n=3) in SMARCA4 mRNA expression (**Figure 1A**).

To explore the role of SMARCA4 in the cell injury response to RSV, hSAECs were stably transfected with lentivirus constitutively expressing control or SMARCA4 directed shRNA. Compared untransfected or control-shRNA transfectants, SMARCA4 shRNA reduced SMARCA4 mRNA to ~25% of control levels (Supporting material, **Figure S1**). Validation of the effect on SMARCA4 protein was performed in Western immunoblot (**Figure 1B**). In WT and CON-shRNA transfectants, SMARCA4 is expressed as a single ~190 kDa protein. By contrast in SMARCA4-shRNA the SMARCA4 abundance is reduced to 17% (Quantitated in **Figure 1C**).

The functional core of mammalian SWI/SNF’s consist of a core ATPase subunit (SMARCA4/Brg1, SMARCA2/BRM) in complex with additional BAF subunits, whose composition is controlled in a cell-type and differentiation-dependent manner (48). Previous work observed that depletion of the SMARCB4/BAF47 subunit produces destabilization and degradation of both the SMARCA4 and SMARCA2 core ATPases through post-translational mechanism (49). To determine the converse, whether downregulation of SMARCA4 affects the abundance of the core proteins, the abundance of SMARC-B1, -E1, and ARID1A were analyzed by Western blot in the SMARCA4-shRNA expressing cells. We observed that SMARCE1 and ARID1A were downregulated >50% by SMARCA4 depletion, whereas SMARCB1 was apparently unaffected (**Figure 1D**). Consequently, we interpret the effect of SMARCA4 shRNA depletion as a depletion of the protein complex of SMARCA4-E1-ARID1A.

### Role of the SMARCA4 Complex on the Genomic Response to RSV Replication

**Figure 2:**
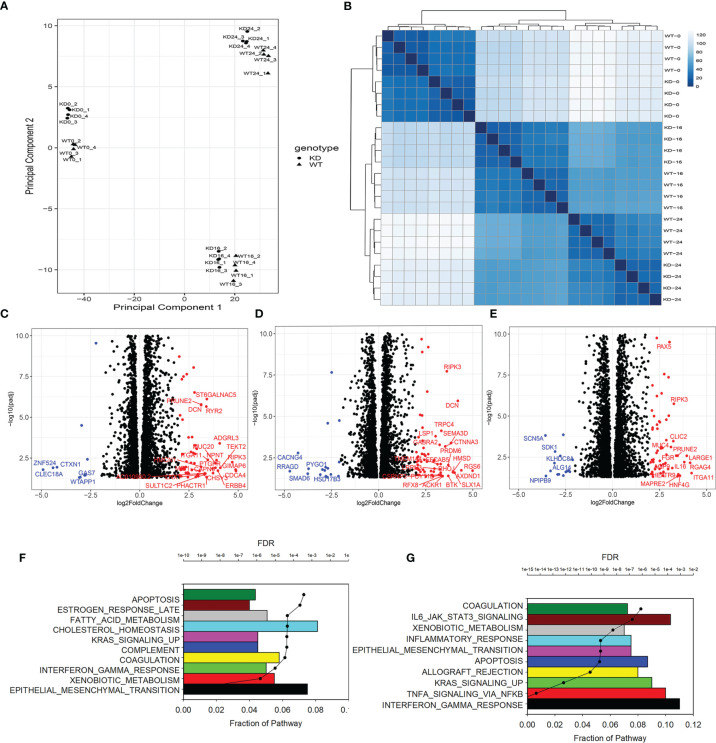
Effect of SMARCA4 complex depletion on RSV-inducible genes. **(A)** Multidimensional scaling of short-read RNA seq. Each point represents independent replicate. KD, SMARCA4 knockdown; WT, wild type. **(B)** Unsupervised clustered image maps of RNA-Seq data. Each row represents an RNA-seq result from CON or RSV infected hSAEC ± SMARCA4-shRNA. **(C)** Volcano plot of differentially expressed genes (DEGs) in uninfected WT vs SMARCA4 KD cells. X axis, log2Fold Change of transcripts/million (TPM). Y axis, -log10(adjusted p value using benjamini-hochberg, padj). **(D)** Volcano plot of DEGs of 16 h RSV-infected WT *vs* SMARCA4 KD cells. **(E)** Volcano plot of DEGs of 16 h RSV infected WT *vs* SMARCA4 KD cells. **(F)** Gene Set Enrichment Analysis (GSEA) of uninfected cells. Genes with 4-fold change in TPM and adjusted p-value of < 0.01 were compared. For each gene set, the fraction of genes represented in the pathway and the significance (false discovery rate, FDR) are plotted. Shown are the top 10 overrepresented pathways. **(G)** GSEA for 24 h RSV-infected cells. Data are shown as above.

RSV replicates in primary human airway epithelial cells, inducing a time dependent global genomic response (50). To examine the effect of the SMARCA4-associated complex on the kinetics of RSV-induced gene expression, SMARCA4-shRNA cells were infected for 0, 16, and 24 h with pRSV (MOI=1; 4 independent replicates of each point). RNA was extracted and subjected to short-read RNA-seq analysis. First global analysis of the expression patterns induced by RSV infection and SMARCA4 complex depletion was performed using an unsupervised approach to visualize sample reproducibility and gene expression similarity of biological treatments. We observed that biological replicates from the same genotype and treatment were closely clustered, indicating minimal biological variance (**Figure 2A**). This biological grouping was reproduced by unsupervised clustered image maps (**Figure 2B**). These data indicate that the global effect on gene expression by SMARCA4 complex depletion was much less than that induced by RSV.

The genomic effects of RSV on hSAEC gene expression have been extensively investigated (50–53). Our focus here is on analysing the effect of SMARCA4 complex depletion. We consequently conducted a series of contrasts using DESEQ2 comparing WT vs SMARCA4 complex depletion for 0 (uninfected), 16 h and 24 h of RSV infection. Here, the –Log10 of the adjusted p-value for the comparison was plotted versus the fold change abundance (transcripts/million) of WT vs SMARCA4 complex-depleted cells. In uninfected cells, 260 genes were expressed at higher levels in the WT cells than in the SMARCA4 complex-depleted cells, whereas a much smaller number (54) genes were expressed higher in the SMARCA4 complex depleted cells (**Figure 2C**). A similar pattern of increased expression was seen in the 16 h and 24 h contrasts (**Figures 2D, E****)**. To identify biological pathways in these differentially expressed genes, gene set enrichment analysis (GSEA) was performed. For uninfected cells, the gene set with the lowest false discovery rate (FDR) is that of the EMT, followed by xenobiotic metabolism, IFN response and others (**Figure 2F**). Consistent with the effects of RSV infection on the innate- pathways, the IFN and NFκB signaling pathways became the most highly upregulated gene sets, although EMT was still in the 10 top-most enriched gene sets (**Figure 2G**).

**Figure 3:**
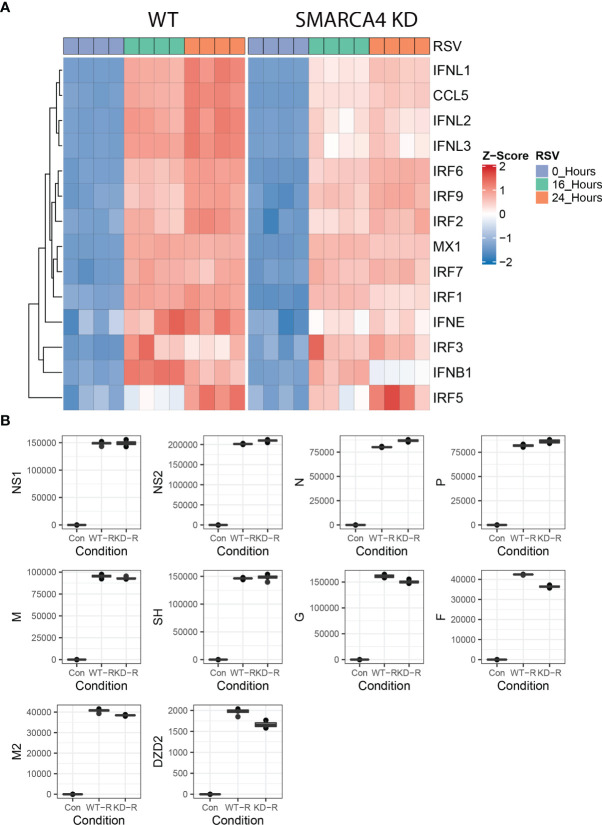
SMARCA4 complex depletion affects type III IFN response without affecting viral transcription. **(A)** Hierarchical cluster of type II and type III IFN genes in WT and SMARCA4 depleted cells. Genes are organized by row, treatment conditions by column. Color indicates row-wise Z-score. Dendrogram is Euclidian distance. **(B)** Quantitation of RSV transcripts. TPM values of short-read RNA seq are plotted by quartile (25%–75%) box plot. Horizontal line is mean value. For each transcript group comparisons are significantly different (2 way-Anova p<0.05) with virus infection *vs* mock-infected (con) contrast p<0.01 post-hoc Tukey’s. DZD2, RNA-dependent RNA polymerase; F, fusion protein; G, glycoprotein; M, matrix protein; N, nucleoprotein; NS, non-structural protein; P, phosphoprotein; SH, small hydrophobic protein.

To further understand the effects of the IFN pathway dysregulation, we identified the subset of IFN regulated genes downregulated in the SMARCA4 complex-depleted cells during the course of the RSV infection, and examined their expression profile by genotype using hierarchical clustering (**Figure 3A**). Here, expression was normalized by Z-score and plotted for each gene in the IFN pathway. We observed IFNL-1, -2, and -3, IFNB1 and the IFN-responsive CCL5 had reduced induction at 16 h and 24 h of RSV infection in the SMARCA4 depleted cells (**Figure 3A**; transcript abundance is shown in **Supporting material**, **Figure S2**).

To determine whether dysregulation of this subset of innate response genes affected RSV replication, the transcripts encoded by this nonsegmented negative-sense RNA virus were individually quantitated in the RNA seq libraries (55). RSV is sequentially transcribed into 10 polyadenylated mRNA species from a 3’ to 5’ direction by its RNA-dependent RNA polymerase in the order 3’ (leader) -NS1-NS2-N-P-M-SH-G-F-M2- DZD2-(trailer) 5’ using a “gene start”- “gene stop” mechanism (55). Consistent with this 3’-5’ gradient of expression, the abundance of NS1 was 75-fold that of low abundance RNA polymerase (DZD2) transcript (**Figure 3B**). Moreover, we found that RSV transcription was essentially unchanged in the SMARCA4 complex-depleted cells vs that of WT, with the exception of slightly increased expression of N, P and decreased DZD2 (**Figure 3B**). Importantly, the IRF3/IFN antagonists, NS1 and NS2 were expressed at indistinguishable levels. Together these data indicated that the SMARCA4 complex was involved in the dynamic IFN response, but this pathway had little overall effect on RSV transcription in this *in vitro* model.

### SMARCA4 Complex Is a Suppressor of the EMT Pathway

**Figure 4:**
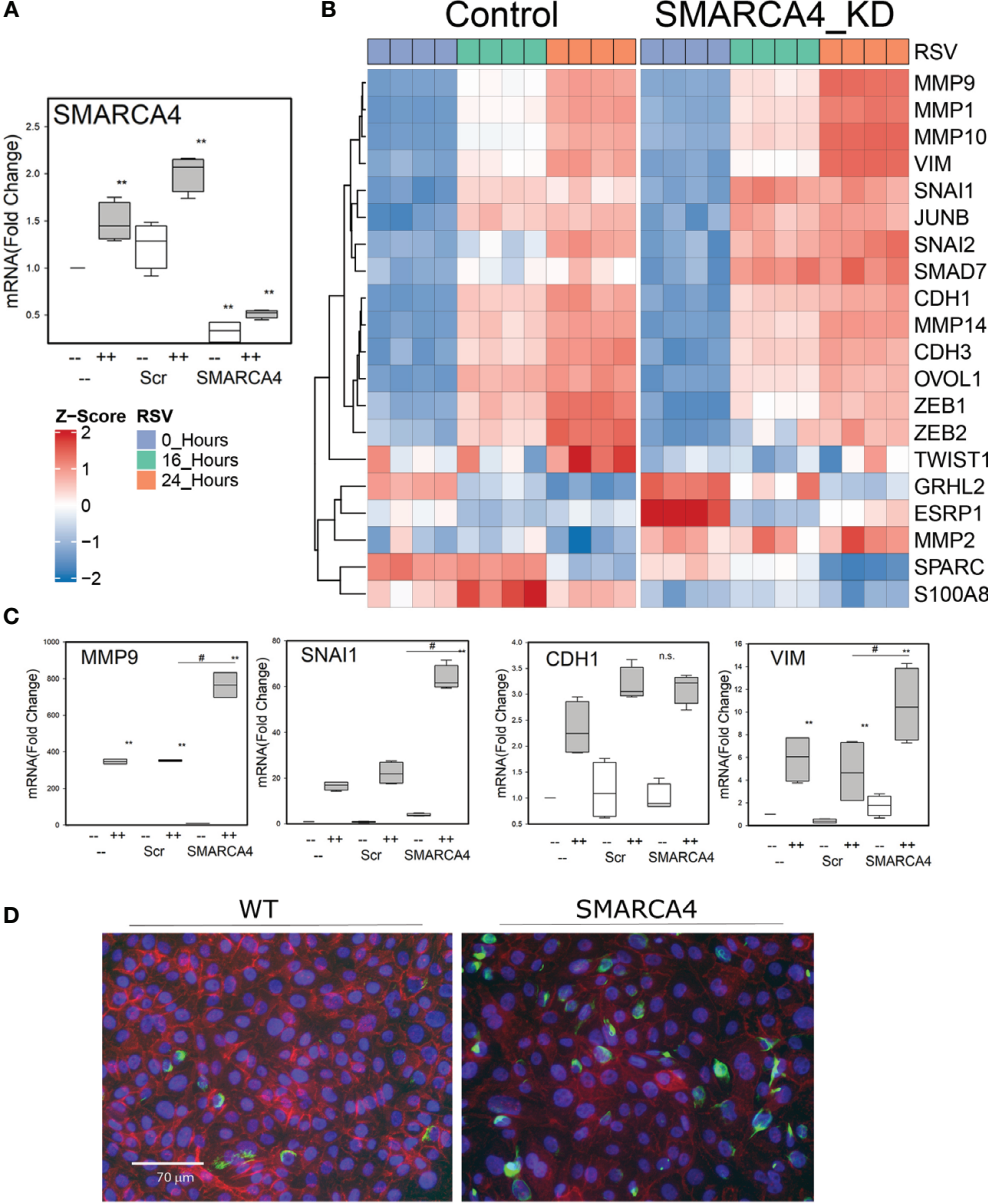
Effect of the SMARCA4 complex depletion on the EMT program. **(A)** Q-RT-PCR for SMARCA4 knockdown. RNA was prepared from uninfected or 24 h infected scrambled or SMARCA4 knockdown cells. Data is normalized to internal control PP1A and expressed as fold change relative to uninfected WT cells. *p <0.01, ANOVA, with contrast using post-hoc Tukeys. **(B)** Hierarchical clustering of EMT signature genes. Expression patterns of signature epithelial or mesenchymal genes in the time course were z-score–normalized and subjected to hierarchical clustering using Euclidian distance. Abbreviations used are: MMP, matrix metalloproteinase; SNAI1, Snail Family Transcriptional Repressor 1; JunB, JunB Proto-Oncogene; CDH, Cadherin; VIM, vimentin; SPARC, Secreted Protein Acidic And Cysteine Rich; ITGB3, integrin Subunit Beta 3; TWIST, Twist Family BHLH Transcription Factor; S100A8, S100 Calcium Binding Protein A8; ZEB: Zinc finger E-box binding homeobox. **(C)** Q-RT-PCR for EMT pathway “signature genes”- *MMP9, VIM*, *SNAI1*, *CDH2*, and *CDH1*. RNA was prepared from uninfected or 24 h infected cells (MOI 1). Data is normalized to internal control PP1A and expressed as fold change relative to uninfected WT cells. **p <0.01, ANOVA, with contrast using post-hoc Tukeys. ^#^p<0.01 compared to Scr. **(D)** Immunofluorescent staining of SMARCA4-depleted cells. Cells were stained with VIM (green) and CDH1 (red). Note the loss of cell surface CDH1 and the accumulation of VIM in the SMARCA4 complex-depleted cells, characteristic of pEMT.

To validate the upregulation of core EMT genes, Q-RT-PCR was conducted in WT, scrambled (Scr) shRNA and SMARCA4 sh-RNA transduced cells in uninfected and 24h infected cells. Confirmation of the selective depletion of SMARCA4 mRNA is shown in **Figure 4A**.

Temporal expression profiling of TGFβ-induced EMT in airway cells has identified sequential waves of epithelial and mesenchymal genes whose expression are important in the manifestation of pEMT and those involved in maintaining stable EMT (23, 29). To determine whether SMARCA4 complex downregulation triggers the EMT response, we examined the expression patterns of these well-established “signature genes” by hierarchical clustering. In uninfected cells, *TWIST, SPARC* and *S100A8* expression were altered in WT vs SMARCA4 complex-depleted cells (**Figure 4A**). Changes in EMT pathway were most notable in response to RSV. For example, in SMARCA4-KD cells, RSV induced expression of *MMPs-1, -2, -9*, and *-10*, *SNAI-1 and-2, JunB*, and *CDH3* at greater levels 16 h after RSV infection (**Figure 4B**; transcript quantitation is shown in the **Supporting Material**, **Figure S3**). At 24 h, a superinduction of *MMPs* -1, -9, and -10, *VIM* and *SNAI*1/2 genes was observed in the SMARCA4 complex-depleted cells 24 h after RSV infection (**Figure 4A**).

Compared to WT and shRNA-Scr transduced cells, SMARCA4 complex depletion dramatically upregulated expression of *MMP9, SNAI1, CDH2*, and *VIM* mRNA (**Figure 4C**). Interestingly, expression the epithelial differentiation marker, *CDH1*, was 2-fold elevated in SMARCA4 complex-depleted cells relative to control cells and slightly induced by RSV infection (**Figure 4C**). Because the expression of the CDH1 epithelial differentiation marker was unaffected, yet mesenchymal and ECM remodeling genes were dramatically upregulated, we conclude that the global effect of SMARCA4 complex depletion was to prime cells to dedifferentiate by inducing a pEMT program.

In light microscopy, we observed that the SMARCA4 complex-depleted cells showed a markedly distinct phenotype from that of control. To demonstrate these changes, WT and SMARCA4-shRNA KD cells were fixed, stained for cytoplasmic VIM and CDH1 and examined by fluorescence microscopy. In control cells, CDH1 was distributed evenly around cellular boundaries, consistent with its role in adherens junction formation (red stain **Figure 4D**). We noted that the abundance of CDH1 staining was reduced in the SMARCA4 complex-depleted cells and, instead of a cell membrane localization, CDH1 staining was predominately cytoplasmic.

Conversely, the numbers of cells expressing cytoplasmic VIM staining was substantially increased in the SMARCA4 complex-depleted cells (**Figure 4D**). The cytoplasmic dyslocalization of CDH1 expression in the context of upregulated expression mesenchymal proteins is further indication that the SMARCA4 complex-depleted cells are in pEMT.

### SMARCA4 Depletion Induces a Metastable State Including Both EMT and MET Programs

**Figure 5:**
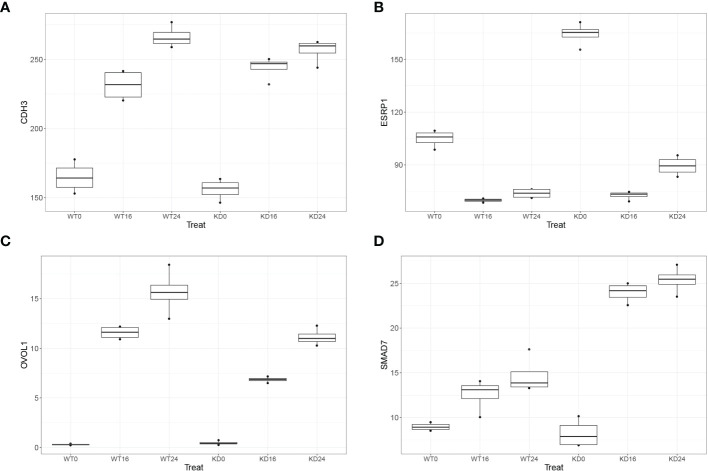
SMARCA4 depletion dysregulates MET programs. Quantitative RNA-seq profiles of key MET regulators are plotted for wild type and SMARCA4 KD cells for 0, 16, and 24 h of RSV infection (all are significant at p<0.05, FDR using DESeq2). **(A)** CDH3; **(B)** ESRP1; **(C)** OVOL1; **(D)** SMAD7.

Plasticity programs in epithelial cell state changes are controlled by gene expression programs that both promote and restrict mesenchymal transition. Expression of key restrictors, such as epithelial splicing regulatory protein 1 (ESRP1), the transcription factor Ovo Like Transcriptional Repressor 1 [OVOL (56)], and Mothers Against Decapentaplegic Homolog 7 [SMAD7 (57)] promote a reverse transition known as MET. Interestingly, we observed that uninfected SMARCA4 complex-depleted cells had increased levels of *ESRP1* expression (**Figure 4B**). To confirm and extend this observation, we examined the quantitative expression of *ESRP1*, *OVOL1*, and *SMAD7* in the WT and SMARCA4-depleted cells (**Figure 5**). We observed that SMARCA4 KD resulted in enhanced expression of *ESRP1* in uninfected cells and was rapidly silenced by RSV infection (**Figure 5**). In contrast, *OVOL1* and *SMAD7* expression were largely unaffected by SMARCA4 complex depletion in uninfected cells, but showed cell-type differences in response to RSV infection. Although RSV-induced *OVOL1* expression was reduced in RSV-infected SMARCA4 KD cells, *SMAD7* expression was dramatically induced by RSV infection (**Figure 5**). Finally, expression of *CDH3*, a marker of hybrid E/M state in cancer cells (58), was highly induced by RSV infection. These data indicate that SMARCA4 plays a role in producing a metastable epithelial cell state with hybrid E/M expression programs.

### SMARCA4 Plays Divergent, Stimulus-Dependent Roles in Chromatin Accessibility

ATAC-seq is an assay that uses hyperactive Tn5 transposase to insert sequencing adapters into accessible regions of native chromatin in intact nuclei, subsequently detected by next generation sequencing. This genome-wide assay has been widely employed to identify nucleosome-free regions of chromatin (34). To determine whether SMARCA4 complex depletion affects the open chromatin landscape, WT or SMARCA4 complex-depleted hSAECs in the absence or presence of pRSV infection (MOI = 1, 24 h) were subjected to ATAC-seq assay. With minimal mitochondrial contamination rate ~25%, an average of 55 M reads were obtained; 95% of the reads were mapped to the human genome, indicating a high degree of genome coverage.

**Figure 6:**
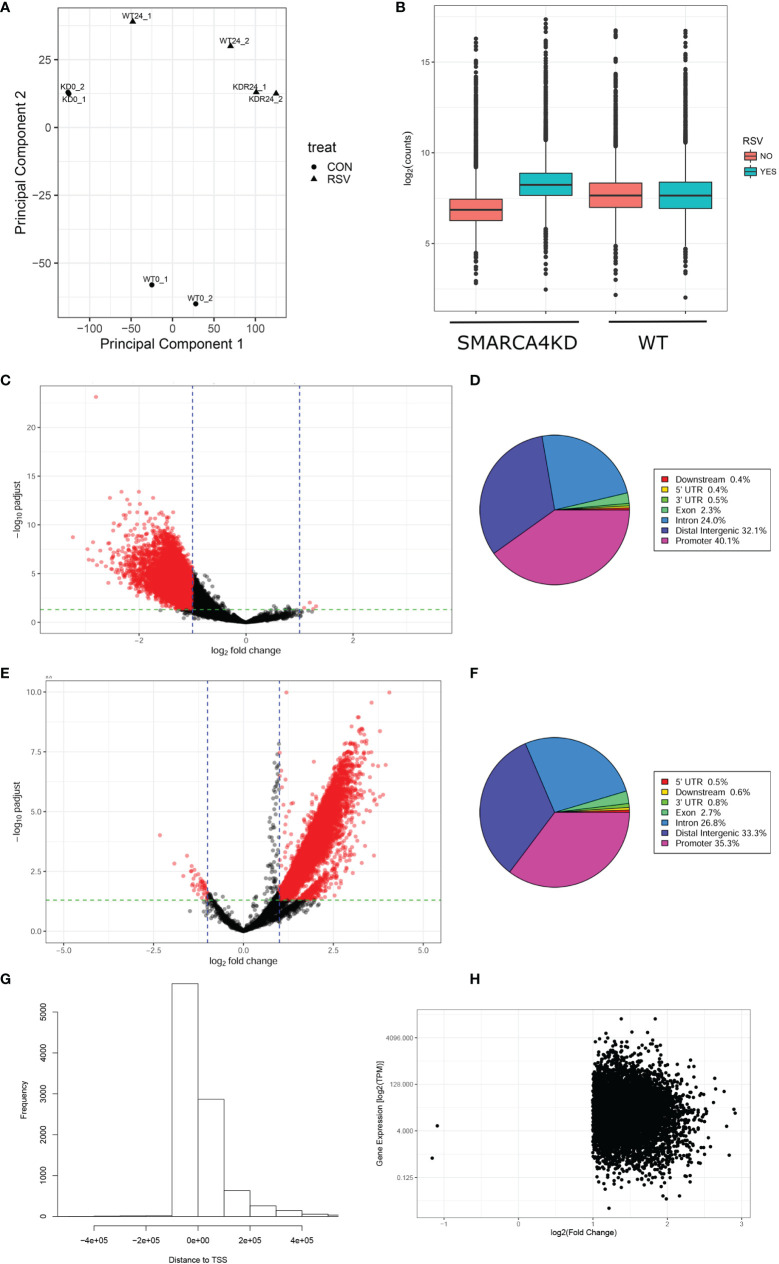
Effects of SMARCA4 complex depletion on chromatin accessibility. **(A)** PCA analysis. PC1 and PC2 explain 76.7% and 13.0% of total variances respectively. **(B)** Distributions of log-transformed count abundance by cell type. **(C, E)** Volcano plots for differentially accessible peaks. **(C)** Uninfected SMARCA4 KD vs. WT cells. **(D)** RSV infection effects between KD and WT. X axis, log2(fold change); Y axis, -log10(adjusted p-value). **(D, F)** Regions of significant ATAC-seq peaks relative to annotated gene bodies. ATAC-seq peaks with 2-fold change and adjusted p value of <0.05 were mapped onto gene bodies (hg19). The distribution of peaks (percent of significant peaks) is shown for each region in the pie chart. Enriched motifs are shown in the **Supporting Material, Figures S3**, **S4**. **(G)** Histogram of location of significant ATAC-Seq peaks relative to gene body annotations. **(H)** Correlation of RSV induced changes in ATAC-Seq peaks in 7,329 promoters with changes in RNA expression (TPM). Note that increases in RSV-induced changes chromatin accessibility are associated with increased gene expression.

A high degree of concordance between experimental replicates for the >27,000 ATAC-seq peaks identified was observed using Principal Component Analysis (**Figure 6A**). Because our focus is on the effects of SMARCA4 complex depletion, differential analysis was performed on significant peaks by DESeq2. Peaks were normalized using sequencing depths to reduce the bias and filtered for a 2-fold change and false discovery rate of 0.05. Reasoning that SMARCA4 regulates open chromatin domains, we compared distribution of the log-transformed ATAC-seq count data across genotypes and treatment conditions. We noted that the uninfected SMARC4 complex-depleted cells have reduced numbers of counts compared to WT cells (**Figure 6B**, Mann-Whitney U p-value < 2.2e-16). Paradoxically, the RSV-infected SMARCA4 complex-depleted cells show greater levels of accessibility in response to RSV infection than WT does (**Figure 6B**, Mann-Whitney U p-value < 2.2e-16).

The contrast comparing genomic accessibility in uninfected cells was further examined using a Volcano plot. For ATAC-seq peaks in WT cells, 34% have reduced cleavage in SMARCA4 depleted cells (**Figure 6C**). The majority of peaks mapped to the promoter (40.1%), intergenic regions (32.1%) and intronic regions of gene bodies (24.0%) (**Figure 5D**). Motif enrichment analysis indicated that transcription factor binding sites for ERF4/SP1, ZNF263, KLF5, TSO1 were enriched in these differential accessible peaks at promoters (**Supporting Material**, **Figure S4**).

The contrast comparing chromatin accessibility fold change in RSV infected cells shows a distinct pattern in the distribution of ATAC-peaks. Among all of the ATAC-seq peaks, 75% of them have a higher fold change in accessibility in SMARCA4 complex-depleted cells than the WT (**Figure 6E**). Binding sites for transcription factors ERF4, OBP3, PUT3, HAP3 were enriched in the RSV inducible SMARCA4 dependent promoter peaks (**Supporting Material**, **Figure S5**). These data indicate that SMARCA4 plays different roles in dynamic changes in chromatin accessibility in infected vs resting cells.

To establish whether changes in chromatin accessibility proximal to annotated genes was associated with increased gene expression on a genome-wide scale, we further examined the relationship between ATAC-Seq peaks occurring in gene promoters and RNA abundance. The distribution of all 13,855 ATAC-Seq peaks affected by RSV interaction relative to gene bodies are shown in **Figure 6F**, where the majority of peaks are located upstream to the regulatory component of the gene. 7,329 significant ATAC-Seq peaks mapped within 2 kb of the transcription start site (**Figure 6G**). Comparison of these peaks with RNA expression indicated that enhanced promoter chromatin accessibility was associated with increased RSV-induced gene expression (**Figure 6H**).

Changes in promoter accessibility does not completely account for the changes in gene expression produced by SMARCA4 complex depletion. For example, detailed analysis of ATAC-Seq cleavage patterns on the IFN genes show no detectable changes on the IFN promoters by SMARCA4 knockdown. However significant changes in extragenic enhancer sequences were observed (**Supporting Material**, **Figure S6**). These findings suggest that SMARCA4 may participate in maintaining conformation of complex chromatin interactions, such as enhancer looping, that will require further study.

### SMARCA4 Regulates Basal and Dynamic Changes in Chromatin Accessibility of EMT Regulators

**Figure 7:**
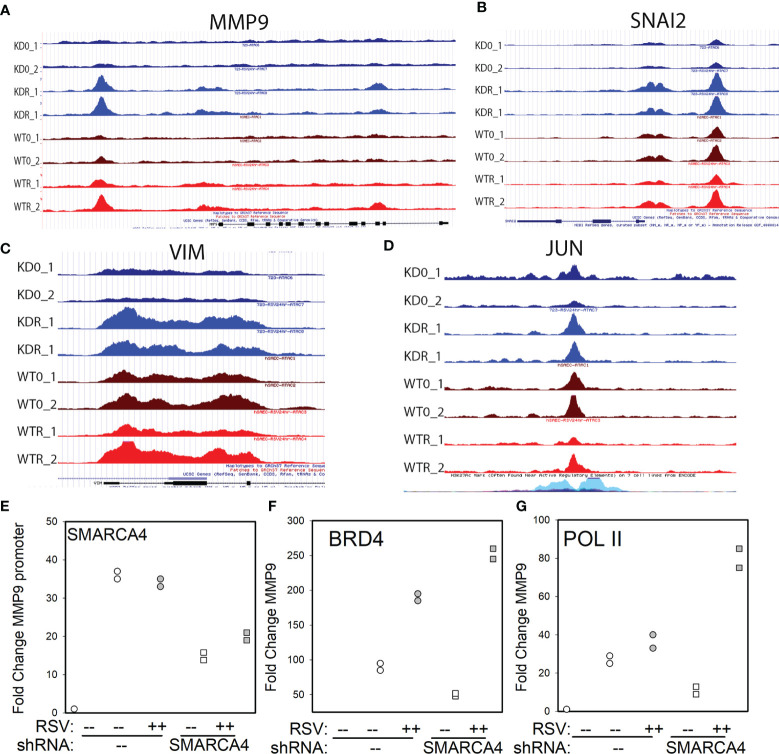
SMARCA4 depletion induces transcription factor loading EMT pathway genes. **(A)** Integrated Genomics Viewer of the individual ATAC seq peaks for uninfected and RSV infected WT and SMARCA4-shRNA KD cells for the MMP9 gene. Each track is shown individually. Location of MMP9 exons and direction of transcription are shown at bottom. **(B)**, IGV of SNAI2 gene. **(C)** IGV of VIM gene. **(D)** IGV view of extragenic enhancer upstream of *JUN*. H3K27Ac peaks, characteristic of an active enhancer are shown at bottom. **(E)** XChIP for SMARCA4 on the *MMP9* proximal promoter. For each IP, Q-gPCR of *MMP9* promoter abundance was performed. Data are presented as fold change over IgG. Individual symbols are biological replicates. Each symbol is the means of technical replicates. **(F)** Q-gPCR of XChIP for BRD4 –long isoform (BRD4-L). **(G)** Q-gPCR for RNA Pol II.

We further examined changes in chromatin accessibility for the pEMT regulatory genes that show exaggerated expression in response to RSV infection. Significant ATAC-seq peaks over the gene loci *MMP9, VIM, SNAI1/2*, and *JUN* were visualized using the Integrated Genomics Viewer (IGV). In uninfected WT cells, *MMP9* has ATAC-seq peaks corresponding to a distal upstream enhancer site and peaks evenly distributed over the promoter and gene body. In response to RSV replication, the accessibility of the *MMP9* upstream enhancer is substantially increased as well as enhanced cleavage on the proximal promoter and gene body, and a more substantial peak over exons 11 and 12 (**Figure 7A**). By contrast in the uninfected SMARCA4 complex-depleted cells, the peak over the *MMP9* upstream enhancer is reduced compared to uninfected WT cells (**Figure 7A**). In response to RSV infection, the cleavage over the upstream enhancer is substantially increased in the SMARCA4 complex-depleted cells relative to that in WT. These data indicate that, paradoxically, the closed chromatin configuration produced by SMARCA4 depletion is highly inducible in response to infection.

A similar pattern of ATAC-seq cleavage patterns are seen for the *SNAI2* gene. In uninfected WT cells, Tn5 transposase cleavage fragments are observed corresponding to an upstream enhancer and the proximal promoter (**Figure 7B**). These are substantially reduced in the uninfected SMARCA4 depleted cells. By contrast, in infected WT cells, the upstream enhancer and proximal promoter demonstrate enhanced cleavage in response to RSV infection (**Figure 7B**). Similarly, in uninfected SMARCA4 complex-depleted cells, the proximal promoter and upstream enhancer of *SNAI2* show reduced accessibility, but are cleaved much more effectively in response to RSV infection. A similar pattern of reduced accessibility in uninfected SMARCA4-depleted cells is seen for the *VIM* gene (**Figure 7C**). A significant extragenic accessibility site was also observed on *JUN* with similar behaviour in response to SMARCA4 depletion (**Figure 7D**).

Collectively, the difference in ATAC-seq peaks (**Figure 6B**), switch from closed- to open chromatin conformations (**Figures 6C–F**) and dynamic changes in individual peak patterns on the pEMT genes indicates that SMARCA4 has divergent-, stimulus-inducible roles in chromatin accessibility and gene repression.

### SMARCA4 Complex Depletion Results in Enhanced BRD4/PolII Loading on MMP9

We focused on understanding further the mechanisms of regulation of *MMP9*, since: 1). ECM remodeling and MMPs are associated with RSV disease severity (59, 60), and 2). MMP9 potentiates EMT *via* SNAI1 (61). To determine whether the changes in chromatin accessibility were associated with distinct loadings of BRD4 and Pol II complex, two-step chromatin immunoprecipitation (XChIP) experiments were conducted. XChIP was used because this method employs a protein-protein crosslinking step to capture protein complexes that may not be directly binding DNA. WT and SMARCA4 complex-depleted cells in the absence or presence of pRSV infection were assayed for SMARCA4, BRD4 and RNA Pol II binding to the proximal *MMP9* promoter. In uninfected WT cells, we observed SMARCA4 binding the *MMP9* promoter, a conclusion supported by the finding that a 36-fold greater signal in MMP9 promoter binding was seen in SMARCA4 immunoprecipitates (IPs) relative to control IgG precipitation in quantitative genomic PCR (Q-gPCR; **Figure 7E**). The *MMP9* promoter binding of SMARCA4 was slightly reduced in WT cells by RSV infection (**Figure 7E**). By contrast, SMARCA4 signal was markedly reduced in SMARCA4-shRNA KD cells in the absence or presence of RSV infection (**Figure 7E**). These data confirm that SMARCA4 directly interacts with *MMP9*.

In uninfected WT cells, BRD4 also binds constitutively to *MMP9*, as demonstrated by 76-fold enrichment in the BRD4 IPs over control IgG (**Figure 7F**). RSV infection induces a further 2.5-fold increase in BRD4 binding to *MMP9*. Notably, in uninfected SMARCA4 complex-depleted cells, basal levels of BRD4 are substantially reduced relative to that of WT controls (**Figure 7F**). And remarkably, in response to RSV infection, BRD4 binding is substantially higher in the SMARCA4 complex-depleted cells than that observed in the RSV-infected WT cells (**Figure 7F**).

A similar pattern of reduced Pol II binding and exaggerated Pol II recruitment was observed in the SMARCA4 complex-depleted cells (**Figure 7G**). These data indicate that SMARCA4 is required for constitutive BRD4/Pol II interaction with *MMP9*, yet plays an inhibitory role for BRD4/Pol II recruitment in response to RSV infection.

### CDH1 Downregulation in SMARCA4 Depletion Is Partially Mediated by MMP9

**Figure 8:**
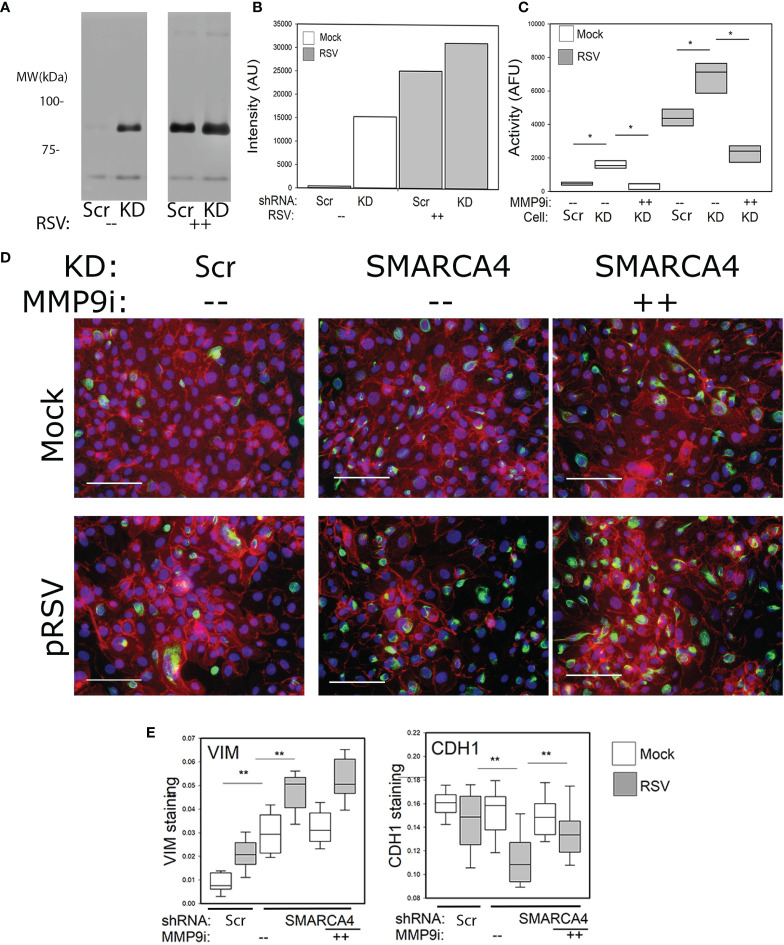
Enhanced cytopathic effect in SMARCA4 complex depleted genes is partially mediated by MMP9. **(A)** In gel zymography of MMP isoforms. Shown is an inverted image of a cell culture supernatant from Scr or SMARCA4 knockdown (KD) cells in the absence or presence of RSV infection (MOI 1, 24 h). After fractionation, proteins were rehydrated and gelatinase activity observed by cleared bands. Approximate migration of molecular weight standards are shown at left. Note the major gelatinase activity is ~90 kDa, corresponding to MMP9. *p<0.05. **(B)** Quantitation of zymogram. Images were analyzed using FIJI and plotted. **(C)** Fluorescent MMP1/9 enzymatic assay. Cell culture supernatants were incubated with MMP1/9 fluorogenic peptide. Fluorescence activity was measured in n=3 lysates. **(D)** Immunofluorescence microscopy. Cells were treated as in **(A)** and stained with CDH1 (red) and VIM (green). Note the dramatic loss of CDH1 in the SMARCA4 complex-depleted cells and increase in VIM. The RSV-induced depletion of CDH1 is reversed by the MM9 inhibitor. **(E)** Quantitation of VIM and CDH1 staining using ImageJ. **p<0.05 Scr vs SMARCA4 shRNA.

Previous work has shown that entry into a hybrid E/M state is mediated by posttranslational regulation of CDH1, where cell surface CDH1 is internalized into endosomal vesicles (11). Our gene expression experiments indicated that RSV induced the expression of a number of MMP isoforms at different levels (**Figure 4B**). To identify the most abundant isoform, gelatin zymograms were performed. Fresh tissue culture supernatants were fractioned by a gelatin-impregnated polyacrylamide gel. Enzymatic activity is renatured, and detected by a zone of clearing. A major gelatinase activity was observed ~90 kDa, indicating that MMP9 was the major metalloproteinase in our system (**Figures 8A, B****)**. To quantitate the enzymatic MMP9 activity we measured MMP9 activity using an MMP1/9 fluorogenic substrate. Consistent with the gene expression changes and zymogram results, uninfected SMARCA4 complex-depleted cells showed higher levels of MM9 enzymatic activity compared to uninfected scrambled (Scr) controls (**Figure 8C**). In both cell types, RSV-induced MMP9 activity by ~4-fold (**Figure 8C**). Finally, we characterized the effect of a potent small molecule MMP9 inhibitor (MMP9 inhibitor I; CAS 1177749-58-4). The MMP9 inhibitor I (5 µM) substantially inhibited MMP enzymatic activity (**Figure 8C**).

To further understand the effect of RSV on pEMT, Scr- or SMARCA4-shRNA transduced cells in the absence or presence of MMP9 inhibitor I were analyzed for VIM and CDH1 expression by immunofluorescence microscopy. In Scr- controls, RSV reduced CDH1 cell-surface expression, resulting in patches of CDH1 negative cells (**Figure 8D**). In SMARCA4 depleted cells, the reduction in CDH1 expression was much more dramatic, producing a more uniform depletion and cytoplasmic distribution (**Figure 8D** middle panel).

In contradistinction to that of CDH1 expression, RSV infection induced slightly the expression of VIM in WT cells, but much more dramatically in the SMARCA4 complex-depleted cells (**Figure 8D**, quantitation in **Figure 8E**).

To determine whether MMP9 participated in cell surface downregulation of CDH1, SMARCA4 complex-depleted cells were treated with MMP9 inhibitor I and analyzed by immunofluorescence microscopy. We noted that MMP9i increased the CDH1 expression and plasma membrane location in response to RSV infection, but had little effect on the VIM expression (**Figure 8E**, right panel). These data indicate that MMP9 partially mediates the CDH1 dysregulation in the absence of SMARCA4.

### SMARCA4 Repression of MMP9 Inhibits RSV-Induced Syncytium Formation

In RSV induced LRTIs viral replication in the distal bronchioles and terminal alveoli induces the formation of multinucleated giant cells (54). Compared to other types of viral infections, including adenovirus, influenza and parainfluenza viruses, giant cell formation appears unique to RSV LRTIs (62). Giant cells play an important role in pathogenesis by forming papillary projections and lower airway obstruction producing the characteristic hyperaeration, V/Q mismatching and hypoxia. To examine the interaction of SMARCA4 and giant cell formation, we tested syncytia formation in the epithelial mesenchymal trophic unit (EMTU). This system preserves the close bidirectional trophic interaction between airway epithelial cells and subepithelial fibroblasts is required for maintenance of the normal airway mucosa and stimulating the injury-repair process (63).

**Figure 9:**
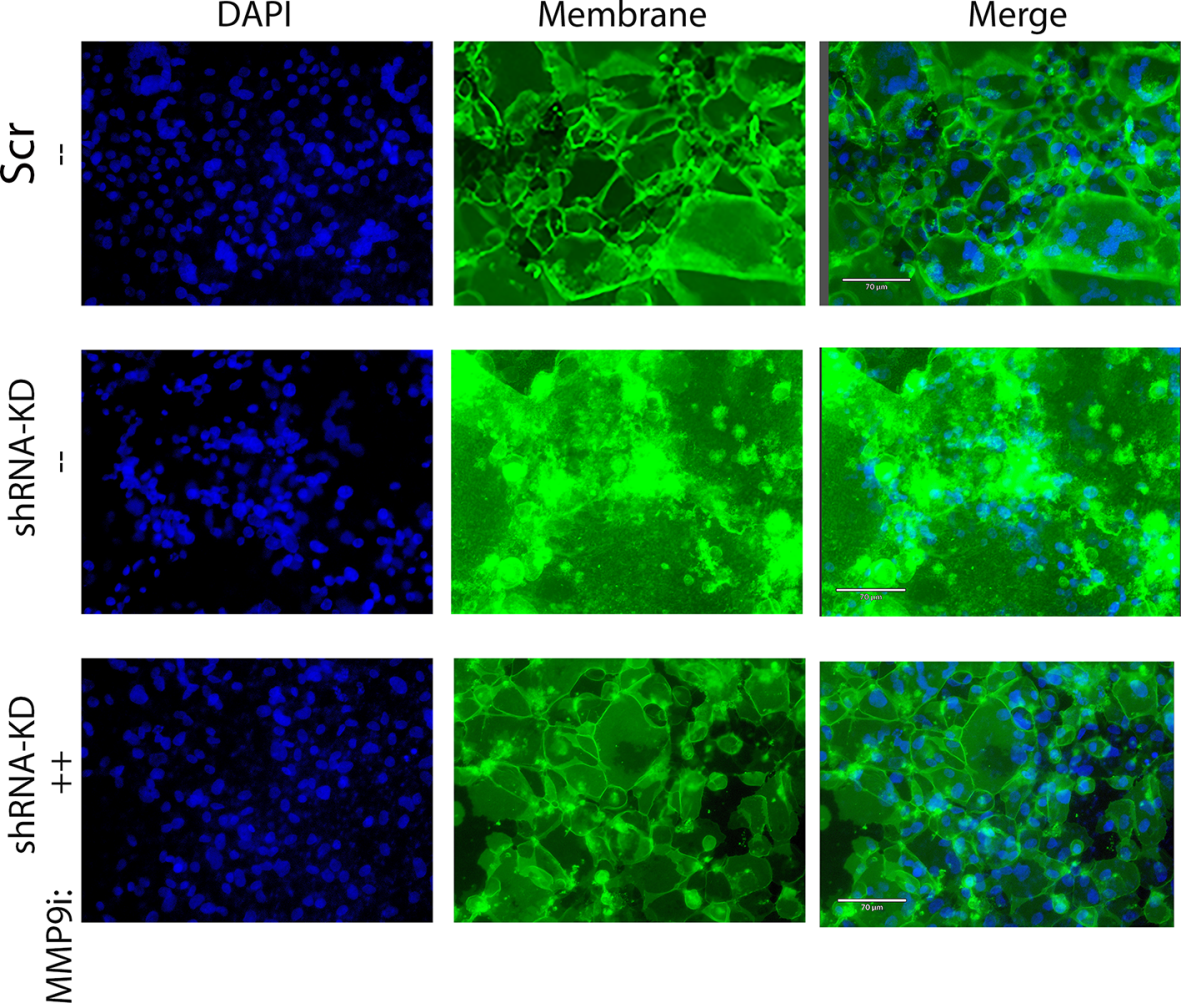
Enhanced syncytia formation by SMARCA4 complex-depleted cells is potentiated by MMP9. To examine responses in a physiological system, where normal epithelial-fibroblast interactions could occur, epithelial cells were co cultured in transwell inserts with submerged NHLFs. Control Scr- or SMARCA4-shRNA cells were infected (pRSV, MOI =0.5, 72 h); SMARCA4-shRNA KD cells were separately treated with MMP9 Inhibitor I (5 µM). Cells were fixed and plasma membrane visualized by staining with CellBright (green) and nuclei stained with DAPI (blue). Note the presence of multiple nuclei within a cellular boundary indicating the formation of a multinucleated giant cell.

To examine syncytia formation, Scr- or SMARCA4 shRNA-transfected cells were cultured in transwell inserts and subjected to RSV infection in the absence or presence of the MMP9 inhibitor I. Cells were fixed then co-stained for cytoplasmic membranes (CellBrite) and nuclei (DAPI). We observed that SMARCA4-shRNA cells showed massive cell fusion, with the entire group of cells losing intercellular boundaries (**Figure 9**).

The RSV fusion (F) glycoprotein precursor is postranslationally processed into active forms by cellular proteases to promote membrane fusion important in viral entry. Remarkably, the exaggerated syncytia formation of the SMARCA4-shRNA KD cells treated with MMP9 inhibitor resembled WT cells (**Figure 9**). These data indicate that SMARCA4 repression of MMP9 modulates RSV-induced giant cell formation.

### SMARCA4 Complex Depletion Results in Enhanced Myofibroblast Formation in the Epithelia Mesenchymal Trophic Unit (EMTU)

**Figure 10:**
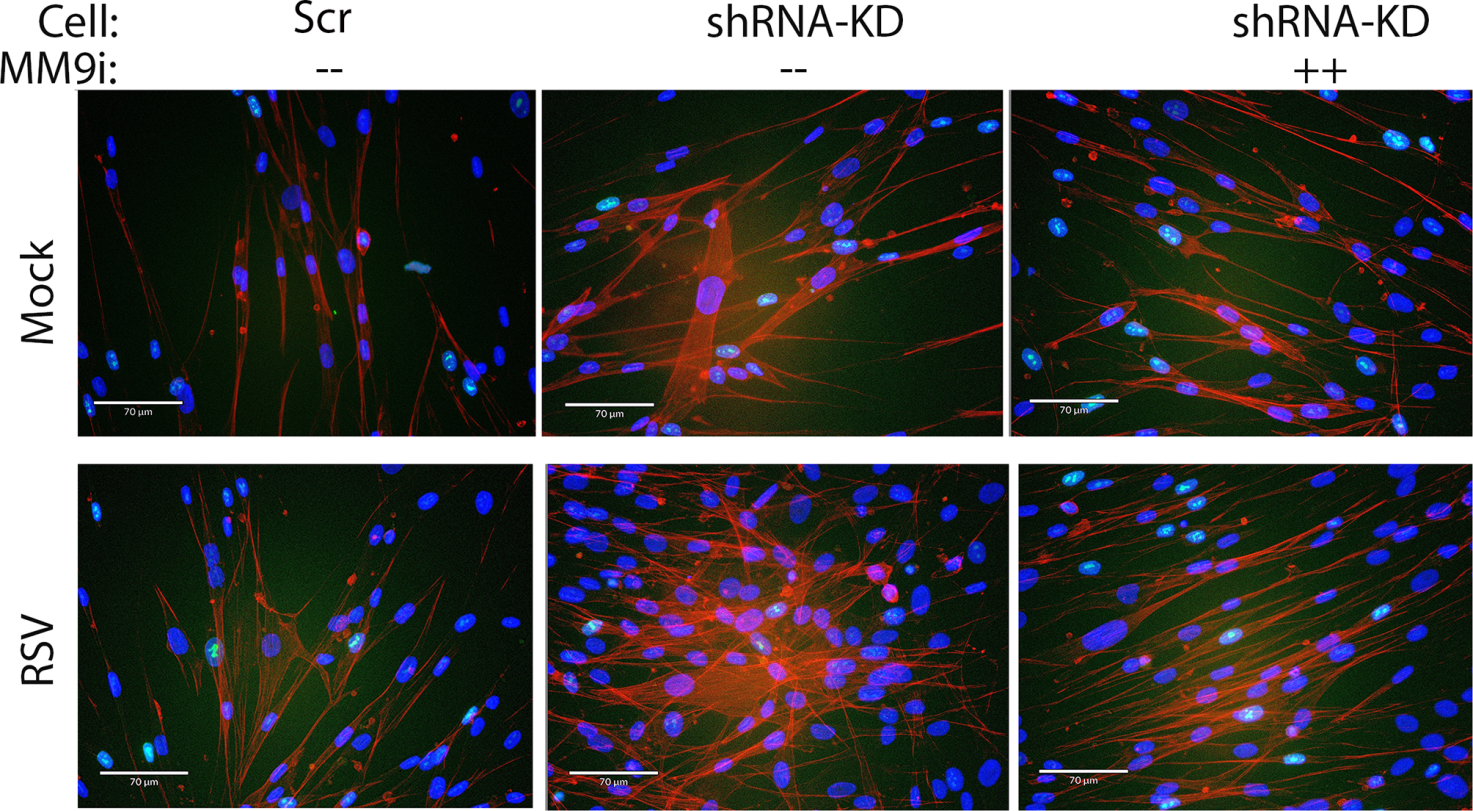
SMARCA4 complex depletion results in enhanced subepithelial myofibroblast formation. Control-Scr shRNA or SMARCA4-shRNA cells cultured in transwell inserts separated from normal human lung fibroblasts (NHLFs) underneath in submerged culture. Eptihelial cells were mock or infected with pRSV (MOI = 0.5, 72 h) in the absence or presence of MMP9 Inhibitor I. NHLFs grown were fixed and stained with phalloidin (red) or DAPI (nuclei) to identify stress fibers. Note the phenotypic change of cell widening and enhanced stress fiber formation in the fibroblasts co-cultured with SMARCA4-shRNA cells and potentiation of stress fiber formation by epithelial RSV infection.

Upon injury epithelial cells release growth factors that induce polymerization of actin fibers (a.k.a. stress fibers) transitioning these fibroblasts into proto-myofibroblasts. Subepithelial normal human lung fibroblasts (NHLFs) from the co-culture experiment (in **Figure 9**) were stained with phalloidin to detect actin stress fibers, indicating myofibroblast transition. In the absence of RSV infection, a greater number of myofibroblasts were formed in the SMARCA4-shRNA coculture vs WT cells (**Figure 10**). In the presence of RSV, slightly greater numbers of myofibroblasts were observed in NHLFs co-cultured with Scr control cells. However, the number of myofibroblasts in NHLFs cultured with RSV-infected SMARCA4 complex-depleted cells were substantially greater. Importantly, the number of myofbroblasts formed in NHLFs cultured with RSV-infected SMARCA4 KD cells were reduced by MMP9 Inhibitor 1 treatment (**Figure 10**). These data indicate that the SMARCA4 complex is a negative regulator of RSV-induced subepithelial myofibroblast formation by inhibition of MMP9.

## Discussion

The airway mucosa is composed of discrete, highly differentiated airway epithelial cells that play important role in gas exchange, maintenance of fluid balance, control of vascular reactivity and initiation of the innate response (1). Specialized cuboidal cells in the lower bronchiolar-alveolar junction are primed to rapidly enter a de-differentiated state to respond to injury produced by aerosolized particulates or respiratory viral infection by rapidly entering EMT and triggering mucosal inflammation (5, 6), processes dependent on the chromatin remodeling and transcriptional elongation activity of BRD4 (64–66). In this study, we examine the dynamic function of the ATP-dependent catalytic kinase, SMARCA4, in RSV induced innate immune responses and epithelial cell state changes. RSV infection is a major human pathogen that activates cuboidal cells of the small airway to induce remodeling, innate activation and Th2 polarization of the airways (6, 67). We discover that SMARCA4 depletion disturbs the steady state abundance of SMARCB1 and ARID1A, SWI/SNF factors known to form a functional complex. RNA-seq studies indicate that SMARCA4 complex plays a role in suppression of an early stage of EMT transition, downregulating cell surface CDH1 and upregulating mesenchymal gene regulatory network including MMP/SNAI/VIM and JUN. Chromatin accessibility studies indicate that SMARCA4 maintains mesenchymal reprogramming genes in an open chromatin configuration. Somewhat paradoxically, upon RSV infection, these genes are dynamically opened, associated with enhanced rates of BRD4 and Pol II loading. Phenotypically, SMARCA4 complex-depleted cells are in pEMT state and undergo dramatically increased formation of multinucleated cells, partially dependent on extracellular MMP9 activity. Moreover, SMARCA4 complex depletion disrupts the EMTU, resulting in enhanced formation of stress-fiber expressing myofibroblasts. These findings indicate that SMARCA4 complex is involved in the terminal differentiation of small airway epithelial cells and plays a dynamic role in controls virus-induced ECM remodeling, myofibroblast expansion, and fibrosis.

### SMARCA4 Regulates Components of the Mucosal IFN Response

Previous work has shown that component of the SWI/SNF complex, BAF47 and its core ATP dependent catalytic subunit SMARCA2/BRM are important in priming IFNβ expression (49). SMARCA2/BRM and SMARCA4/BRG1 are nonredundant ATP-dependent chromatin remodeling complexes that are functionally independent. Interestingly, here, we observe that SMARCA4 complexes are also important in priming both type II (IFNβ), and type III (IFNL) mucosal interferons for a full anti-viral response. Our studies suggest that this priming effect may be due to dysregulation of upstream IRFs, critical transcriptional activators of IFNβ/L pathways. Interestingly, SMARCA4 complex depletion does not directly affect IFN promoter accessibility but rather, distal upstream enhancer/intergenic elements. These findings suggest that SMARCA4 may be involved in maintain 3D chromatin conformation and enhancer looping, questions that will require independent investigation. Previous work has shown that IRFs are regulated by changes in epithelial cell state, where TGFβ induced EMT silences expression of the IRF1 pathway (14). More work will be needed to understand whether effect on IFN expression is a direct or indirect effect and its consequences in more complex anti-viral responses.

### SMARCA4 Depletion Is Associated with Enhanced Epithelial Mesenchymal Plasticity

Detailed mechanistic and computational work exploring the findings that cells express both epithelial and mesenchymal factors has led to the conclusion that EMT is not simply a binary transition from epithelial to mesenchymal states, but rather a series of multiple transitions with various degrees of hybrid epithelial/mesenchymal (E/M) states (10). These states (and the distribution of cell populations within them) are governed by stochastic processes and are both stimulus and context-dependent. A spectrum of epithelial-mesenchymal plasticity has been proposed that transitions relevant for cancer- these include populations with various degrees of invasiveness, proliferation, cell cycle regulation, some of which are probably not relevant transitions for untransformed epithelial cells.

Nevertheless, our study does support an extensive phenotypic characterization of the effects of SMARCA4 depletion. For example, the loss of CDH1 and cell-cell contacts (**Figure 4D**) indicates that the hybrid E/M state induced by SMARCA4 complex depletion results in loss of adherence and mucosal tight junctions. The reduction in IFN production indicates that the hybrid E/M state affects anti-mucosal innate immunity (**Figure 3A**). Our data demonstrate SMARCA4 deficient cells have enhanced secretion of biologically active collagenases that promote ECM remodeling and tissue invasion (**Figure 8**). Finally, the cells are producing paracrine factors that promote myofibroblast transition (**Figure 10**). These data indicate that SMARCA4 controls a multi-dimensional phenotypic response important in airway biology, repair and response to viral infection.

### SMARCA4 Promotes a Metastable Hybrid E/M State

Here, we observe that depletion of the SMARCA4 chromatin remodeling complex transitions hSAECs into metastable state characterized by expression of epithelial and mesenchymal differentiation regulators. Detailed time-series studies of TGFβ-induced EMT indicate that stable EMT is the result of sequential expression of gene expression networks under control of autoregulated master transcription factors. A first step involves loss of the cell surface CDH1 *via* a post-transcriptional mechanism involving CDH1 internalization (11) is a reversible stage where cells can revert back to normal epithelium (MET) or transition to stable EMT depending on cellular context and cues (10, 12, 68). Of note, this hybrid E/M state is characterized by loss of differentiated epithelial markers (CDH1), expression of EMT repressors, and expression of mesenchymal genes (23, 29). We note that CDH1 mRNA expression is maintained in SMARCA4 depleted cells, yet the protein is mislocalized and rapidly depleted in response to RSV infection, consistent with other studies in cancer. The absence of CDH1 mRNA silencing may be due to the lack of ZEB expression; others have shown that ZEB represses CDH1 by recruitment of SMARCA4 (69). Collectively, our study suggests that SMARCA4 complex is an important regulator of epithelial cell plasticity. One notable difference is that

Previous work shows that SMARCA4 is required in coordinating the TGFβ -induced EMT (70). Here, SMARCA4 was found to complex with activated SMAD3 an association required to recruit to activate downstream genes, including CTGF *via* Pol II recruitment. These results are in apparent contradiction to our studies, where SMARCA4 depletion induces unscheduled entry into pEMT. It is well-established that the composition of the SMARCA4 complex is cell type dependent and dynamically reconfigured by changes in differentiation state (71). The explanation for this different roles, then, may be due to cell type differences in the composition of the complex. We interpret our studies to mean that SMARCA4 is an epithelial cell identity gene, preventing small airway epithelial cells from entering pEMT by default.

Our recent demonstration that tonic activation of the innate pathway results in EMT driven by the master transcription factor, NFκB/RelA (23), is highly relevant to our finding that RSV triggers expression of core mesenchymal transcription factors. Our earlier work showed that expression of the hallmark mesenchymal transcription factors, SNAI1/ZEB/TWIST, are all amplified by NFκB by binding their upstream promoters (23, 64, 72). Here we observe that in WT hSAECs, RSV induces a cascade of EMT regulatory factors (JunB, JunD, SNAI), and that RSV-induced expression of this regulatory factor clique is enhanced by SMARCA4 complex depletion. Our implication that SMARCA4 functions as an epithelial differentiation specification gene is consistent with the findings that SMARCA4 is required for maintenance of skeletal muscle differentiation by associating with MyoD and Mef2 to activate myogenic-specific gene promoters (73).

### Repressive Activity of the SMARCA4 Complex

Work in yeast originally showed that SMARCA4 is an ATP-dependent multisubunit complex that modifies nucleosomal structure resulting in enhanced chromatin accessibility (74). In mesenchymal cells, SWI/SNF antagonizes the repressive roles of Polycomb repressive complexes (PRCs). In our studies, ATAC-seq cleavage patterns suggest that chromatin accessibility for the promoters of key pEMT drivers (JUN, SNAI1/2) is reduced in the absence of SMARCA4 complex, yet expression of these genes is higher in SMARCA4 complex-depleted cells. To us, these data suggest that SMARCA4 may function as a suppressor by reducing chromatin accessibility but the enhanced BRD4 and Pol II recruitment associated with enhanced expression is difficult to explain. In other cell contexts, SMARCA4 functions as a suppressor by binding histone deacetylases, the transcriptional co-repressors mSinA and the nuclear corepressor (NCoR) (75). Finally in SMARCA4-depleted mammary epithelial cells, HiC and ChIP-Seq studies indicate that SMARCA4 plays a role in maintaining higher-order genome organization and superenhancer formation controlling expression of extracellular matrix genes (76). More work will be required to identify this complex interactions between nuclear organization, chromatin accessibility and corepressor interactions.

MMP9 is a highly regulated gene controlled by step-wise interactions of corepressor and activator complexes binding to an upstream control region. In HeLa carcinoma cells, MMP9 expression is activated by phorbol esters that induce MEK-1-and NFκB, whose binding stimulates release of corepressor complexes that constitutively bind MMP9 in unstimulated cells (77). Corepressors identified in this study include Sin3A/HDAC1 and nuclear co repressor (NcoR)/HDAC3; both complexes are known to bind SMARCA4 (75). These data further suggest the intricate relationship between chromatin accessibility and corepressor recruitment mediate SMARCA4 inhibition of MMP9 and other pEMT genes.

### MMP9 and Its Role in RSV Infection

In this manuscript we focus on MMP9 and its cellular effects in response to RSV infection. MMP9 is tightly regulated neutral gelatinase that degrades extracellular matrix (ECM) proteins, facilitating lung inflammation and activating innate inflammation (defensins) (78). Because excessive MMP activation is associated with destructive lung disease, its regulation is tightly controlled at the expression level and through production of tissue inhibitor of metalloproteinases (TIMPs). Previous work has shown that increased activities of ECM- remodeling proteins have been associated with severity of RSV disease (60), including MMP9 (79). In fact, dysregulated MMP9 is a marker of airflow obstruction and diminished lung function in obstructive lung diseases (80). Interestingly MMP9 activity induces cytoskeletal reorganization and SNAI expression (61) and conversely, SNAI expression induces MMP9 (81). These suggest SNAI-MMP9 are in an amplification loop controlling EMT.

### MMP9 Mediates RSV Induced Syncytia Formation

Multi-nucleated giant cells are hallmarks of the cytopathic effect of RSV implicated in airway obstruction in severe disease (54). Pathological findings of RSV LRTIs include the presence of multinucleated giant cells (54). Compared to other types of viral infections, including adenovirus, influenza, and parainfluenza viruses, giant cell formation appears unique to RSV LRTIs (62). Syncytia formation is one mechanism for facilitating cell-cell spread of virus, a process in lower respiratory tract spread observed in human challenge studies (46). We observe that depletion of the SMARCA4 complex produces a dramatic cell fusion response to RSV infection and that this response is reduced by MMP9 inhibition. These data are consistent with the findings that cells expressing higher levels of MMP9 form greater numbers of syncytia, and inhibited by TIMP expression (82). Syncytia formation is induced by the RSV F protein that mediates cellular entry. Following docking to the plasma membrane, RSV is internalized by an actin-dependent endocytotic pathway where RSV F1 undergoes a second cleavage reaction (83). This cleavage is mediated by cellular furins; however this is also an MMP9 recognition site. Activated F forms a trimeric structure that results in membrane fusion (84). A prediction for future experimentation is that greater abundance of activated RSV F is found in SMARCA4 complex-depleted cells.

### MMP9 Plays a Role in Paracrine Activation of Myofibroblast Transdifferentiation

A central effector cell for airway fibrosis is the myofibroblast, a pleiotropic mesenchymal-derived cell involved in the excessive deposition of extracellular matrix in the lamina reticularis. Myofibroblasts are a highly dynamic population whose number increase in response to viral infections. Our early studies indicate that viral pattern-induced mesenchymal transition may play an important role in myofibroblast transition though incompletely understood mechanisms (66). A close bidirectional interrelationship exists between epithelium and myofibroblasts. In response to injury, epithelial cells secrete paracrine growth factors that may enhance formation of myofibroblasts. However, the role of MMP9 in viral induced myofibroblast expansion has not been explored.

Earlier studies indicate that secreted MMP9 binds to receptors on fibroblasts to activate α-smooth muscle actin (α-SMA) expression and myofibroblast transition (85). Our intriguing studies show that viral induced MMP9 expression is a major paracrine regulator of myofbroblast transdifferentiation. These findings provide new mechanistic linkages between virus induced epithelial injury and remodeling.

In summary, our studies provide a novel role for the SMARCA4 regulator as an epithelial differentiation factor, suppressing mesenchymal transition, and controlling activation of the MMP9 locus in the fibrotic response to RSV infection. Inhibition of MMP9 could reduce epithelial cell damage and fibroblast transdifferentiation. It also worth noticing that enhancing the expression of SMARCA4 in lung airway epithelial cells could be a potential therapeutic target.

## Data Availability Statement

The datasets presented in this study can be found in online repositories. The names of the repository/repositories and accession number(s) can be found below: https://www.ncbi.nlm.nih.gov/genbank/, GSE161849.

## Author Contributions

Experimentation: XX, DQ, and AB. Analysis XX, CD, DQ, AB, MM, and SK. concept XX, SK, RG, and AB. Writing XX, DQ, CD, MM, KS, RG, and AB. All authors contributed to the article and approved the submitted version.

## Funding

This work was partially supported by NIH grants AI062885 (AB, RG) and NCATS UL1TR002373 (AB). The funders had no role in the design of the study; in the collection, analyses, or interpretation of data; in the writing of the manuscript, or in the decision to publish the results.

## Conflict of Interest

The authors declare that the research was conducted in the absence of any commercial or financial relationships that could be construed as a potential conflict of interest.
