# Supplementary material for: The SWI/SNF-Related, Matrix Associated, Actin-Dependent Regulator of Chromatin A4 Core Complex Represses Respiratory Syncytial Virus-Induced Syncytia Formation and Subepithelial Myofibroblast Transition
Source: Front Immunol. 2021 Mar 1;12:633654. doi: 10.3389/fimmu.2021.633654 (PMC7957062; doi:10.3389/fimmu.2021.633654)

Supporting Material

Figure S1. shRNA knockdown of SMARCA4. Q-RT-PCR of control, scrambled (Scr) shRNA expressing cells and SMARCA4 shRNA expressing cells for SMARCA4 mRNA. Box plots are +/-25% quartile of n=4 independent assays. Data is presented as fold change relative to control cells. **, $p<0.05$, two-tailed t test.

Figure S2. Quantitation of IFN expression. RSEM values (counts normalized to million base pairs) for indicated genes for WT or SMARCA4 shRNA (KD) cells as a function of RSV infection. Box plots are +/-25% quartile of n=4 independent assays.

Figure S3. Quantitation of EMT regulatory genes. RSEM values (counts normalized to million base pairs) for indicated genes for WT or SMARCA4 shRNA (KD) cells as a function of RSV infection. Box plots are +/-25% quartile of n=4 independent assays.

Figure S4. Motif enrichment for downregulated peaks. Enriched motifs identified in downregulated ATAC-Seq peaks (WT vs KD contrast). Shown are ERF4/SP1, ZNF263, KLF5, TSO1.

Figure S5. Motif enrichment for upregulated peaks. Enriched motifs identified in upregulated ATAC-Seq peaks (WT vs KD contrast). Shown are ERF4/SP1, OBP3, PUT3, and HAP3.

Figure S6. Effect of SMARCA4 depletion on intergenic IFNB1 and IRF1 enhancers. A, Integrated Genomics View of ATAC-seq peaks in KD and WT cells upstream of *IFNB1*. B, IGV of intergenic locus of IRF1 gene.

Figure S1.

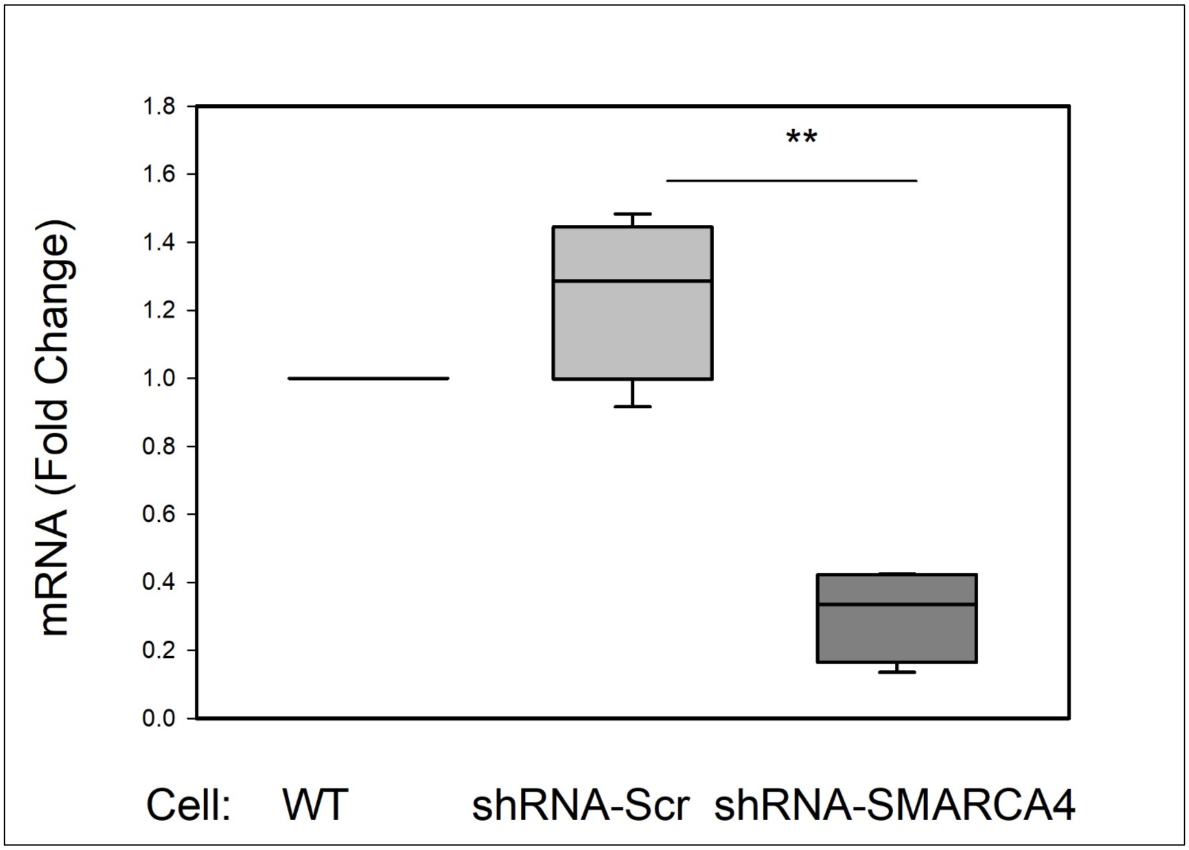
1.8
1.6
1.4
1.2
1.0
0.8
0.6
0.4
0.2
0.0
mRNA (Fold Change)
**
Cell:
WT
shRNA-Scr
shRNA-SMARCA4

Figure S2.

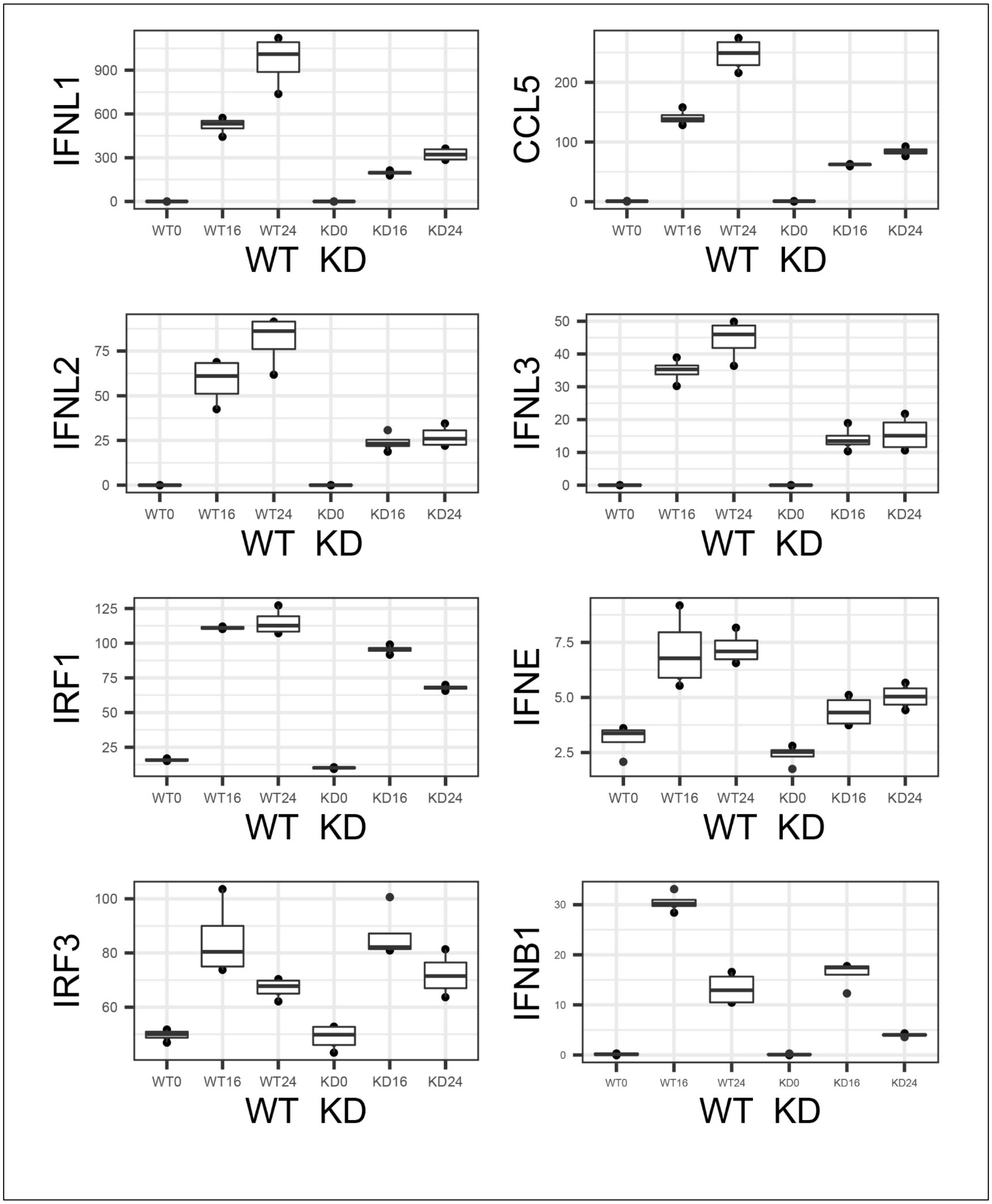

IFNL1
CCL5
IFNL2
IFNL3
IRF1
IFNE
IRF3
IFNB1
WT0 WT16 WT24 KD0 KD16 KD24
WT KD

Figure S3.

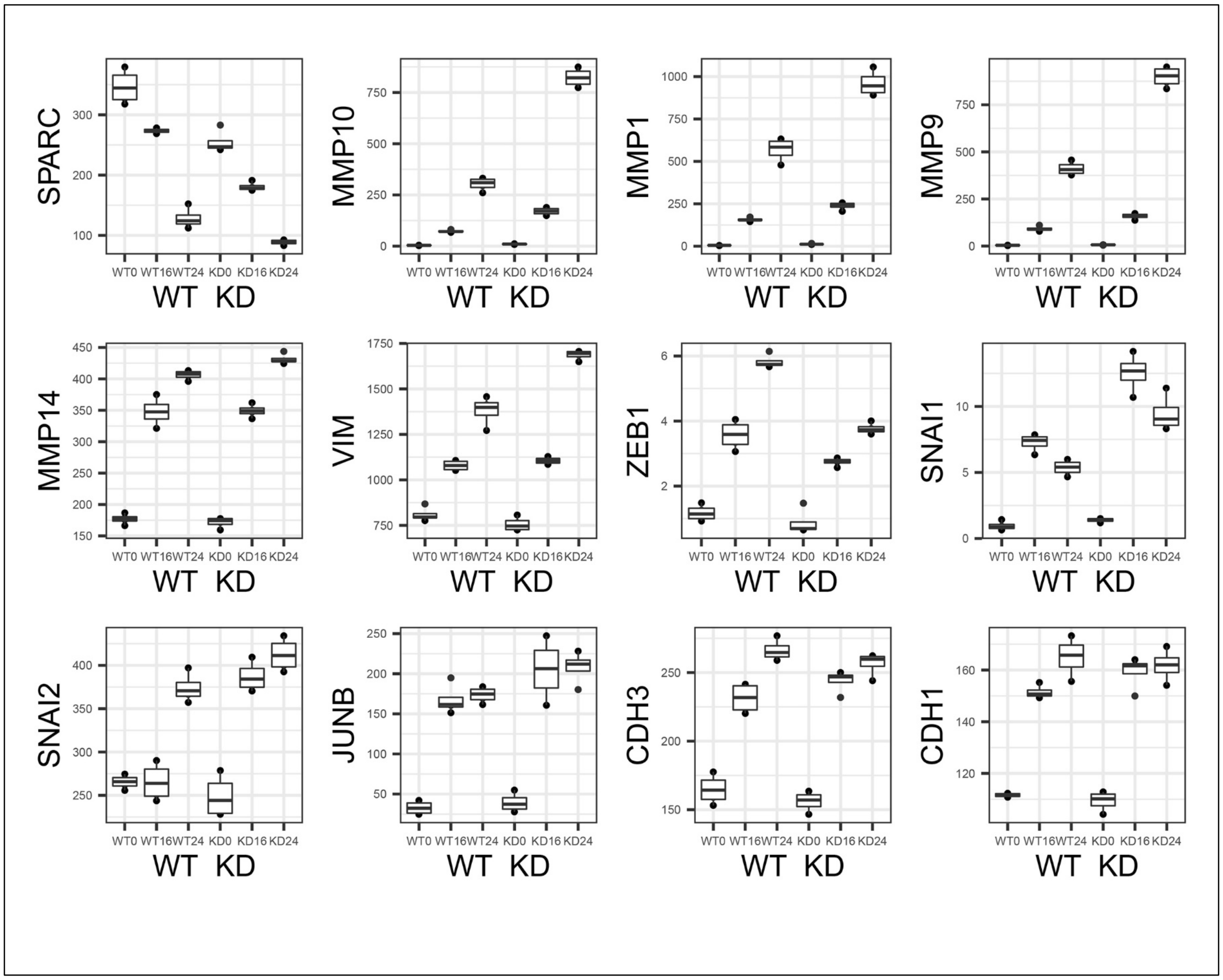
SPARC
MMP10
MMP1
MMP9
MMP14
VIM
ZEB1
SNAI1
SNAI2
JUNB
CDH3
CDH1
WT0 WT16 WT24 KD0 KD16 KD24
WT KD

Figure S4

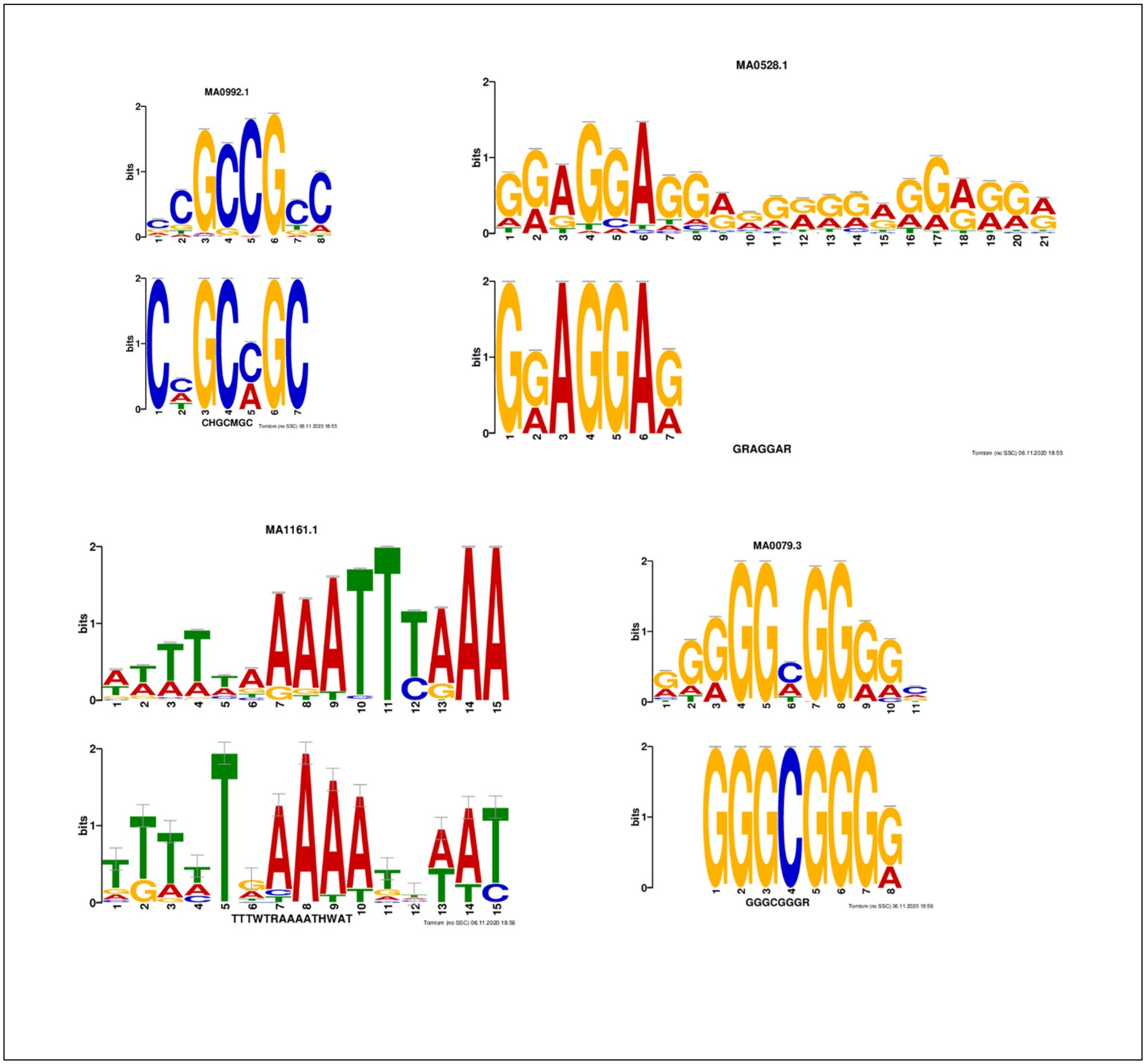
MA0992.1
CHGCMGC
MA0528.1
GRAGGAR
MA1161.1
TTTWTRAAAATHWAT
MA0079.3
GGGCGGGR

Figure S5

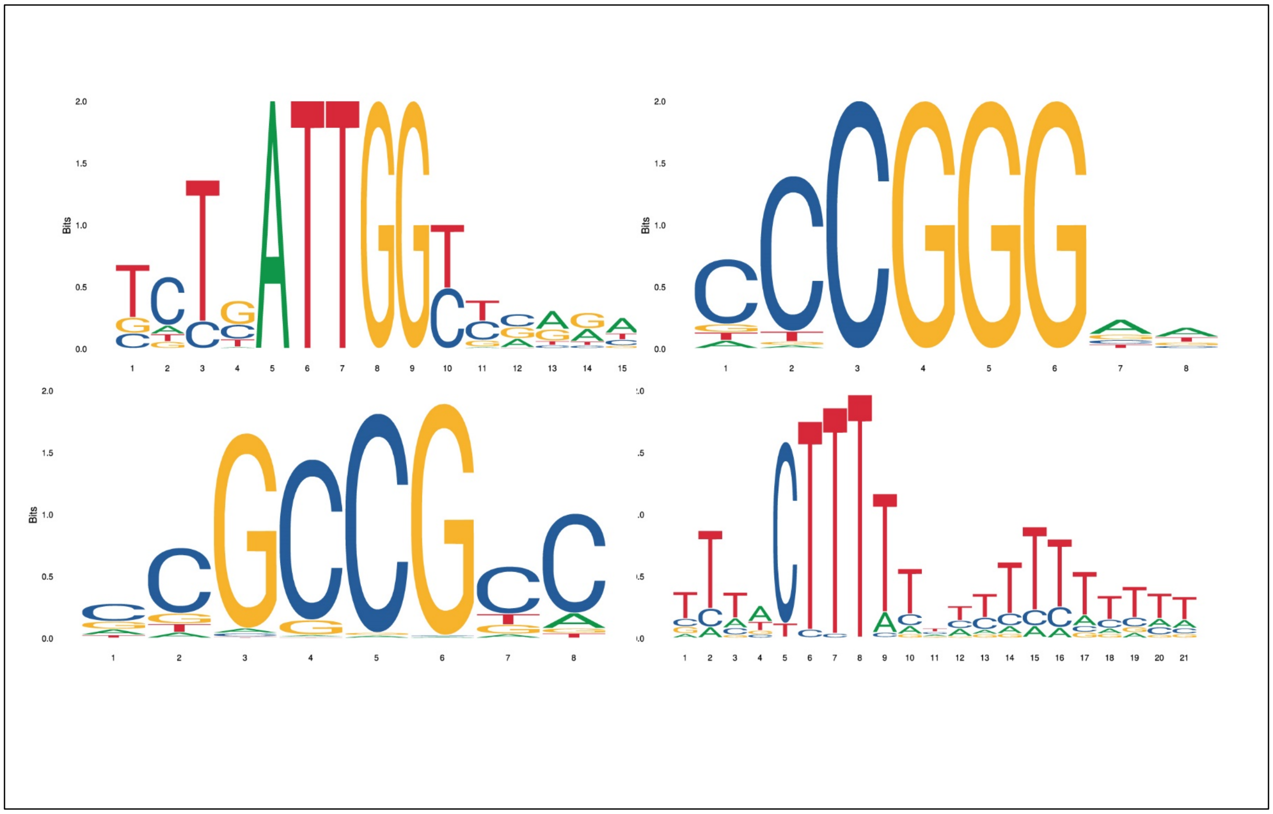

Figure S6A.

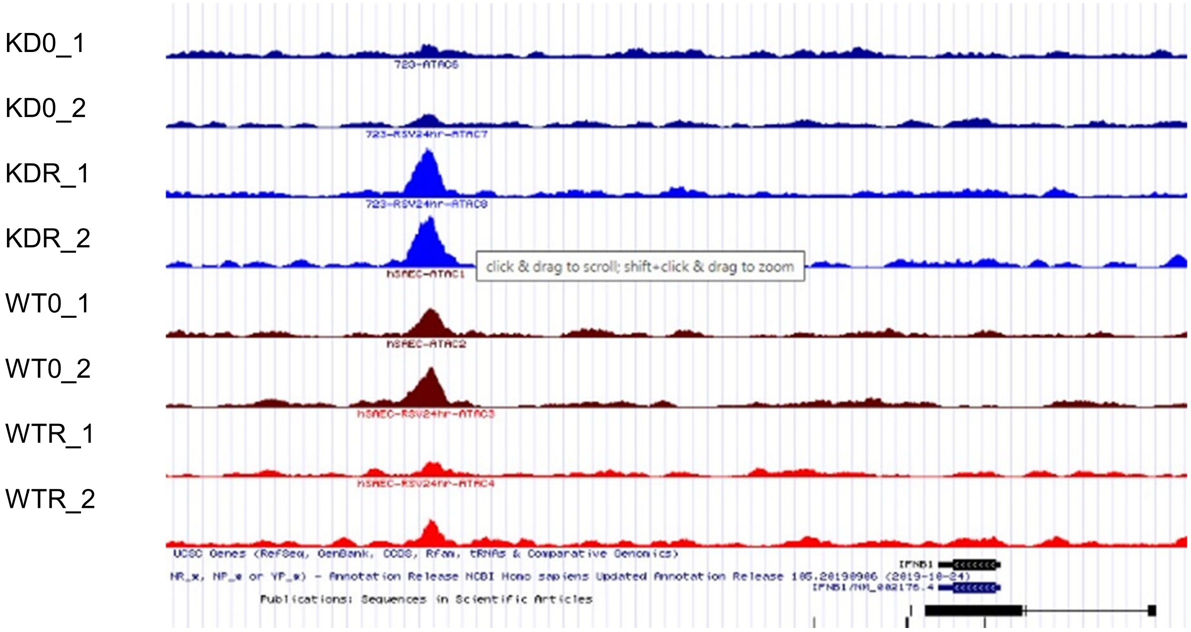

Figure S6B.

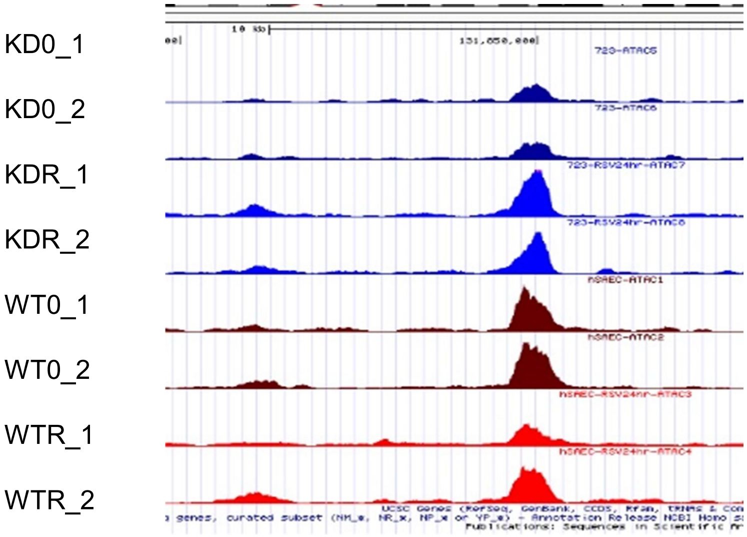

Supplement: Supplementary file 1 [file DataSheet_1.pdf]
